# Cortical oxygen extraction fraction using quantitative BOLD MRI and cerebral blood flow during vasodilation

**DOI:** 10.3389/fphys.2023.1231793

**Published:** 2023-10-06

**Authors:** Linh N. N. Le, Gregory J. Wheeler, Emily N. Holy, Corinne A. Donnay, Nicholas P. Blockley, Alan H. Yee, Kwan L. Ng, Audrey P. Fan

**Affiliations:** ^1^ Department of Biomedical Engineering, University of California, Davis, Davis, CA, United States; ^2^ Department of Neurology, University of California, Davis, Davis, CA, United States; ^3^ School of Medicine and Health Sciences, University of Nottingham, Nottingham, United Kingdom

**Keywords:** Oxygen extraction fraction (OEF), cerebral blood flow (CBF), vasodilation, arterial spin labeling (ASL) MRI, quantitative BOLD (qBOLD), magnetic resonance imaging (MRI)

## Abstract

**Introduction:** We aimed to demonstrate non-invasive measurements of regional oxygen extraction fraction (OEF) from quantitative BOLD MRI modeling at baseline and after pharmacological vasodilation. We hypothesized that OEF decreases in response to vasodilation with acetazolamide (ACZ) in healthy conditions, reflecting compensation in regions with increased cerebral blood flow (CBF), while cerebral metabolic rate of oxygen (CMRO_2_) remained unchanged. We also aimed to assess the relationship between OEF and perfusion in the default mode network (DMN) regions that have shown associations with vascular risk factors and cerebrovascular reactivity in different neurological conditions.

**Material and methods:** Eight healthy subjects (47 ± 13 years, 6 female) were scanned on a 3 T scanner with a 32-channel head coil before and after administration of 15 mg/kg ACZ as a pharmacological vasodilator. The MR imaging acquisition protocols included: 1) A Gradient Echo Slice Excitation Profile Imaging Asymmetric Spin Echo scan to quantify OEF, deoxygenated blood volume, and reversible transverse relaxation rate (R_2_^’^) and 2) a multi-post labeling delay arterial spin labeling scan to measure CBF. To assess changes in each parameter due to vasodilation, two-way *t*-tests were performed for all pairs (baseline versus vasodilation) in the DMN brain regions with Bonferroni correction for multiple comparisons. The relationships between CBF versus OEF and CBF versus R_2_’ were analyzed and compared across DMN regions using linear, mixed-effect models.

**Results:** During vasodilation, CBF significantly increased in the medial frontal cortex ( P=0.004 ), posterior cingulate gyrus (pCG) ( P=0.004 ), precuneus cortex (PCun) ( P=0.004 ), and occipital pole ( P=0.001 ). Concurrently, a significant decrease in OEF was observed only in the pCG (8.8%, P=0.003 ) and PCun ( 8.7%,P=0.001 ). CMRO_2_ showed a trend of increased values after vasodilation, but these differences were not significant after correction for multiple comparisons . Although R_2_’ showed a slightly decreasing trend, no statistically significant changes were found in any regions in response to ACZ. The CBF response to ACZ exhibited a stronger negative correlation with OEF ( β=−0.104±0.027 ; t=−3.852,P<0.001 ), than with R_2_’ ( β=−0.016±0.006 ; t=−2.692,P=0.008 ).

**Conclusion:** Quantitative BOLD modeling can reliably measure OEF across multiple physiological conditions and captures vascular changes with higher sensitivity than R_2_’ values. The inverse correlation between OEF and CBF across regions in DMN, suggests that these two measurements, in response to ACZ vasodilation, are reliable indicators of tissue health in this healthy cohort.

## 1 Introduction

The brain has a high metabolic demand for oxygen compared to other organs since the human brain comprises just 2% of the total body mass but consumes 20% of available oxygen for normal function (Rolfe and Brown, 1997), so regulation of cerebral blood flow (CBF) and oxygen delivery is critically important. Oxygen extraction fraction (OEF) is a key hemodynamic parameter to measure the brain’s energy metabolism and is altered in aging and disease processes, including cerebrovascular disorders (Watchmaker et al., 2018; Fan et al., 2022; Liu et al., 2023), neurodegeneration (Jiang et al., 2020; Robb et al., 2022), and neuroinflammation (Cleland et al., 2021). In some pathological mechanisms, OEF is reduced concurrently with hypoperfusion due to decreased neural activity and lower oxygen metabolic demand (Ishii et al., 1996; Brown and Thore, 2011); while in other distinct mechanisms, OEF is elevated in areas of hypoperfusion, indicating physiological compensation in tissue that is at ischemic risk (Gupta et al., 2014; Fan et al., 2020; Lin et al., 2022). Therefore, it is important to measure OEF in tandem with CBF to understand overall oxygen consumption and brain tissue health.

In addition to resting state, imaging brain hemodynamics in varying physiological conditions, such as those created by controlled vasoactive stimuli, adds valuable information about cerebrovascular reactivity (CVR) and brain vascular health. These cerebrovascular “stress tests” monitor the CVR, or the brain’s hemodynamic response to hypercapnic gas paradigms or to pharmacological vasodilation, e.g., with acetazolamide (ACZ) (Handwerker et al., 2007). CVR measurements reveal unique pathophysiological changes in intracranial stenosis (Lattanzi et al., 2018) and in aging that correlate with cognitive status (Silvestrini et al., 2006; Kim et al., 2021). During these vasoactive challenges, brain hemodynamics are typically monitored with the blood-oxygenation-level-dependent (BOLD) MRI signal due to its fast acquisition and ease of use; however, BOLD signal changes reflect multiple neuronal and vascular contributions and cannot be interpreted in the context of a specific hemodynamic parameter. Other investigations have used arterial spin labeling (ASL) MRI to directly assess the perfusion reactivity deficits in steno-occlusive disease and validate the BOLD response in the same patients (Mandell et al., 2008; Smeeing et al., 2016), highlighting ASL as a robust biomarker to quantify CBF in different physiological states and disease. Acetazolamide has been commonly used to assess the vasodilatory capacity of circulation because ACZ is more easily administered through intravenous or oral means than carbon dioxide, which requires a gas delivery system. In patients with major cerebral arterial occlusive diseases, ACZ also identified reduced reactivity in the hemisphere with the occlusive lesion even in patients who appeared to have preserved CO_2_ reactivity (Kazumata et al., 1996). However, few studies have directly assessed OEF changes during vasoactive stimuli, largely because of a lack of non-invasive imaging approaches. [^15^O]-PET scans have measured CBF changes in response to ACZ vasodilation in patients with cerebrovascular stenoses and showed a non-linear association between this perfusion reactivity and baseline OEF PET for various ischemic disease stages (Imaizumi et al., 2004; Nemoto et al., 2004; Yamauchi et al., 2004; Hokari et al., 2008). Unfortunately, these OEF PET measures are not easily available in different physiological states due to the complexity of the experiments and the need to administer multiple short-lived radiotracers for each condition. MRI-based OEF measures have recently been tested with ACZ administration and showed expected global OEF reductions after vasodilation using multiple T_2_ relaxation-based measures (Baas et al., 2022). Venous blood oxygenation (Y_v_), which can be derived from venous blood T_2_ relaxation values in the sagittal sinus, combined with global cerebral metabolic rate of oxygen, has shown poorer oxygen utilization in patients with sickle cell disease compared to controls after vasodilation (Václavů et al., 2020), albeit with some bias between various T_2_-based MRI methods. Decreased OEF after ACZ vasodilation has also been demonstrated using 3-dimensional quantitative susceptibility mapping MRI (Buch et al., 2017). However, these values were averaged from larger, resolvable internal cerebral veins, thus limiting regional information on OEF response across the brain.

As an alternative to global OEF measurements using T_2_ relaxation-based approach, quantitative blood-oxygenation-level-dependent (qBOLD) is a non-invasive MRI approach that models the temporal signal due to reversible transverse relaxation rate (R_2_’) to non-invasively extract OEF and deoxygenated blood volume (DBV) (Yablonskiy and Haacke, 1994). This method allows for voxelwise estimation of tissue OEF from asymmetric spin echo (ASE) scans, which can be useful in identifying areas of the brain that may be at risk for ischemia in stroke (Stone et al., 2019) or sickle cell disease (Guilliams et al., 2018; Wang et al., 2021). These clinical studies used qBOLD to identify regional, individualized pathophysiology in different ischemic tissue types (core, infarct growth, and contralateral tissue) in acute stroke. On the other hand, in patients with sickle cell anemia, abnormal OEF elevations were observed specifically in the deep white matter, consistent with microstructural damage, that were reduced post-blood transfusion. Additionally, OEF activation has been reported to decrease in brain areas relating to motor task execution (Yin et al., 2018), especially under hypoxic conditions (Yin et al., 2021). In aging, studies have identified CVR reductions in brain regions that overlap with the default mode network (DMN), which is related to underlying vascular risk factors and suggests CVR in the DMN-CVR to be an important marker for brain health (Haight et al., 2015). Additional investigation of the reliability of qBOLD MRI in sensitive brain areas, including the DMN, across physiological states is thus critical to advance regional OEF as an informative biomarker in cerebrovascular disease and aging.

This study aimed to demonstrate a quantitative BOLD modeling MRI method for measuring OEF values in healthy participants during a physiological perturbation with ACZ. Previous studies have shown that OEF decreases in response to an increase in CBF from various vasodilating challenges, while CMRO_2_ remains unaffected using different techniques (Kety and Schmidt, 1948; Vorstrup et al., 1984; Václavů et al., 2020; Vestergaard et al., 2023). We aimed to demonstrate this method by showing that OEF measured with qBOLD MRI is directly related to regional alterations in CBF. A secondary aim was to compare regional OEF with R_2_’ values in detecting an alteration with vasodilation and their correlation with perfusion. Furthermore, we focused on assessing the OEF relationship to perfusion in the DMN due to its functional associations with vascular risk factors (Tchistiakova et al., 2015) and cerebrovascular reactivity in cognitive impairment (Richiardi et al., 2015) to identify which DMN regions show the most significant OEF effect with vasodilation.

## 2 Materials and methods

### 2.1 Study population

Eight healthy subjects (47 ± 13 years, 6 female) were recruited and gave written informed consent to participate in the study. Participants must be at least 18 years old to participate in this study. Exclusion criteria included pregnancy, anemia, history of renal disease, hypertension, diabetes, stroke, or other known neurological diseases. Subjects with peripheral vasculopathy or Raynaud’s disease that precludes IV administration and a history of allergy to sulfa drugs were excluded. In addition, participants that could not receive MRI due to the inability to lie motionless in the scanner, pacemakers, aneurysm clips, neurostimulators, artificial heart valves, metal objects in eyes, ear implants, and claustrophobia were excluded. This study was performed with approval from the Institutional Review Board at the University of California, Davis.

### 2.2 Vasodilatory stimulus

After baseline MRI scanning, all participants were given a slow administration dosage of 15 mg/kg ACZ as a pharmacological vasodilator over 2 min. After at least 15 min of uptake time to capture the full effect of pharmacological vasodilation, the participant received repeated scans in the post-ACZ state.

### 2.3 MRI acquisition

Each participant underwent scanning on a 3-T MRI scanner (Siemens MAGNETOM Tim Trio, Erlangen, Germany) with a 32-channel phase array head coil. T_1_-weighted anatomic images were acquired using a multi-echo magnetization-prepared rapid gradient echo (MEMPRAGE) sequence before vasodilation. These sagittal scans were acquired with repetition time (TR) = 2530 ms; echo times (TE) = 1.64, 3.5, 5.36, and 7.22 ms; inversion time (TI) = 1,200 ms; spatial resolution = 1.0 mm^3^ × 1.0 mm^3^ × 1.0 mm^3^; in-plane matrix = 256 × 256; 176 slices with thickness = 1.0 mm; flip angle = 7.0°; and acquisition time of 6 min and 2 s.

For OEF measurements, the Gradient Echo Slice Excitation Profile Imaging (GESEPI) ASE images were acquired with an echo planar imaging (EPI) readout with specialized z-shim gradients mitigating through-slice inhomogeneities (Blockley and Stone, 2016). Scan parameters included TR = 3,000 ms; TE = 56 ms; slice thickness = 1.25 mm; resolution = 2.3 mm^3^ × 2.3 mm^3^ × 1.25 mm^3^; flip angle = 90°; bandwidth = 2,004 Hz/Pixel; matrix size = 96 × 96 with 80 slices; field of view (FOV) = 224 mm with 7 values of the spin echo displacement time (τ) from 16 to 40 ms in the step of 4 ms. A total of 20 slabs were acquired, and each slab was constructed by averaging four 1.25-mm slices to correct for macroscopic field gradients with 100% partition oversampling (total 8 k-space partitions). The total acquisition time of the main ASE sequence was 2 min and 54 s to cover the whole brain with 80 slices. A separate spin-echo ASE scan (*τ* = 0) was collected with the same scan parameters as described, with an acquisition time of 24 s.

To generate quantitative CBF maps, we performed multi post-labeling-delay (PLD) 3D pseudo-continuous ASL (pcASL) MRI. The parameters were five different PLDs (0.9 s, 1.2 s, 1.4 s, 1.8 s, and 2.1 s) and effective labeling durations = 2 s; TR/TE = 4,700 ms/14 ms; slice thickness = 4 mm; spatial resolution = 3.4 mm^3^ × 3.4 mm^3^ × 4.0 mm^3^; FOV = 220 mm (Li et al., 2015). Multi-band EPI readout was used with slice-acceleration factor of 6, FOV shift factor of 3, and EPI factor of 64. Including calibration scans with similar imaging parameters but no labeling to estimate longitudinal magnetization (M_0_), the total acquisition time was approximately 6 min. The ASE and pcASL scans were acquired twice—once at baseline and once after vasodilation.

### 2.4 Image analysis

#### 2.4.1 Image preprocessing

Prior to qBOLD modeling, ASE data were preprocessed first with motion correction of all echoes to the spin echo volume using *MCFLIRT* (Jenkinson et al., 2002). Next, the spin echo image was brain extracted using *BET* (Smith, 2002) to create a binary mask of the brain tissue, which was applied to all ASE R_2_’-weighted images. ASE data were finally smoothed with a Gaussian kernel (σ = 4 mm) in FSL (Jenkinson et al., 2012) to reduce the effect of noisy voxels. The empirical tradeoff between spatial resolution and noise reduction is shown in Supplementary Figure S4.

For pcASL data, images from all five PLDs were merged and underwent the same preprocessing steps as the ASE data. First, motion correction was applied with reference to the first pcASL image, followed by smoothing with a Gaussian kernel of 1.5 mm. Then, the M_0_ image was brain extracted using *BET* to create the binary mask of the brain tissue, which was used for registration.

#### 2.4.2 OEF quantification

##### 2.4.2.1 Two-compartment model theory for quantitative BOLD

Quantitative measurements of the BOLD signal were used to non-invasively map hemodynamic parameters relating to brain metabolism and function. The quantitative BOLD (qBOLD) model (Yablonskiy and Haacke, 1994) measures oxygen extraction fraction from the measured reversible transverse relaxation rate, R_2_’ (where R_2_’ = R_2_*–R_2_). A complete qBOLD model with R_2_’-weighted measurements can be achieved using asymmetric spin echoes (An and Lin, 2003), such as the GESEPI-ASE pulse sequence described above. qBOLD modeling was performed with two compartments (tissue and blood), as this approach provides reliable estimates of OEF with a full range of echoes for fitting, including those before the spin echo (Cherukara et al., 2019).

The objective of qBOLD modeling is to separate OEF and DBV effects from the ASE signal and generate a brain map of OEF. The (1) tissue signal, (Yablonskiy and Haacke, 1994), $S^{t}$ is:

$$S^{t}\left(\tau \right)=\left\{\begin{array}{cc} S_{0}\exp\left(-R_{2}^{t}\times TE\right)\times \exp{\left(-\frac{3}{10}DBV\left(\delta \omega \times \tau \right)\right)}^{2} & \left|\tau \right|<T_{c}\\ S_{0}\exp\left(-R_{2}^{t}\times TE\right)\times \exp\left(DBV–DBV\times \left(\delta \omega \times \tau \right)\right) & \left|\tau \right|>T_{c} \end{array}\right.$$ (1)

where δω is the characteristic frequency (with $\left.R_{2}^{\prime }=DBV\times \delta \omega \right)$ , *t*_*c*_ is the characteristic time, and $R_{2}^{t}$ is the irreversible transverse relaxation rate of bulk tissue. The R_2_’-weighted signal has different behavior in two different regimes of the parameter τ (Eq. 1). The boundary between these regimes is considered to be at T_c_ = 1.76t_c_ (Cherukara et al., 2019). The characteristic time $t_{c}$ was defined as $t_{c}=\frac{1}{\delta \omega }$ (Lee et al., 2018). The time variable τ corresponds to the spin echo displacement values of the ASE acquisition. The (2) blood signal, $S^{b}$ *,* (Sukstanskii and Yablonskiy, 2001; Yablonskiy et al., 2013) is:

$$S^{b}\left(\tau \right)=\exp\left(-R_{2}^{b}\times TE+i\frac{\delta \omega \times \tau }{2}\right)\frac{C\left(\eta \right)-iS\left(\eta \right)}{\left(\eta \right)}$$ (2)

where *C(* η *)* and *S(* η *)* are Fresnel function, and $\eta ={\left(\frac{3\delta \omega \left|\tau \right|}{\pi }\right)}^{2}$ . $R_{2}^{b}$ is the transverse relaxation rate of blood and is described as a function of the fractional hematocrit (*Hct*) and OEF of the intravascular compartment (Simon et al., 2016):

$$R_{2}^{b}=\left(16.4Hct+4.5\right)+\left(165.2Hct+55.7\right)\times OEF^{2}$$ (3)

The total signal calculated from a voxel in this two-compartment tissue model is the sum of the signal from each compartment:

$$S^{Total}\left(\tau \right)=S_{0}\left(DBV\times S^{b}\left(\tau \right)+\left(1-DBV\right)\times S^{t}\left(\tau \right)\right)$$ (4)

After fitting the two-compartment tissue qBOLD model, R_2_’ and DBV were estimated. Then, OEF was determined by the relationship between R_2_’ and DBV with known constants:

$$OEF=\frac{3\times R_{2}^{\prime }}{4\pi \times \gamma B_{0}\times ∆\chi_{0}\times Hct\times DBV}$$ (5)

where *Hct* is the patient’s fractional hematocrit, *Δχ*_*0*_ is the magnetic susceptibility difference between oxygenated and deoxygenated red blood cells, *B*_*0*_ is the magnetic field, *γ* is proton gyromagnetic ratio.

R_2_’-weighting was acquired by shifting the spin echo refocusing pulse for 7 values of the spin echo displacement time, *τ*, in the step of 4 ms from 16 ms to 40 ms. The spin echo (*τ* = 0 ms) was collected in separate scan. *Hct* was assumed at 0.40 (Nicoll et al., 2012). Other physiological parameters were also set as constant values as follows: $∆\chi_{0}=0.264\times 10^{-6}$ (Spees et al., 2001); $R_{2}^{t}=11.5s^{-1}$ (He and Yablonskiy, 2007). $R_{2}^{t}=11.5s^{-1}$ . Additionally, acquisition parameter values used for the model were $B_{0}$ = 3T, TE=56ms , and TR=3s.

##### 2.4.2.2 Bayesian inference for mapping OEF with qBOLD

**FIGURE 1:**
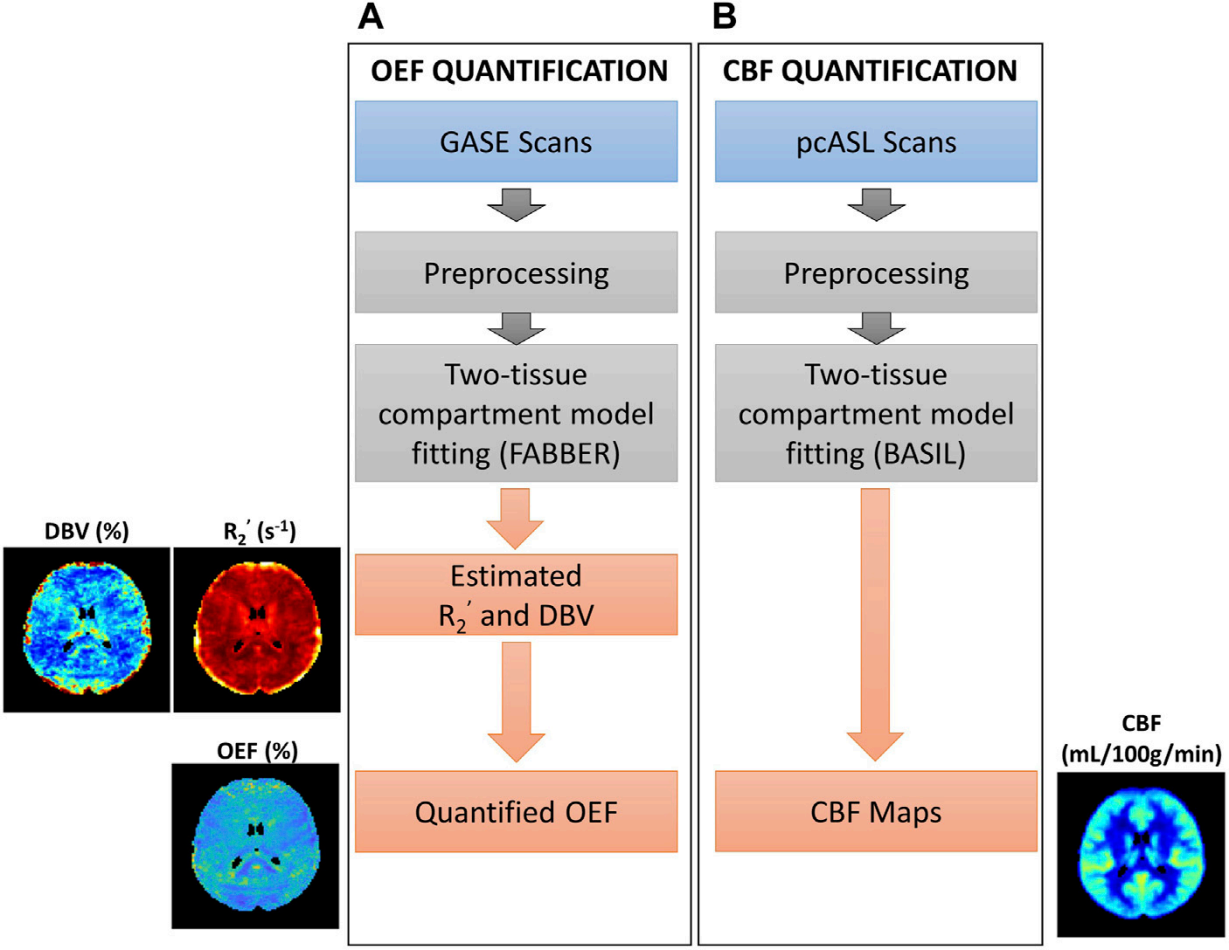
Overview of procedures for parameter quantification. **(A)** Oxygen extraction fraction (OEF) quantification using GASE scans was achieved through fitting with two-tissue compartment qBOLD model in FABBER (Woolrich et al., 2006; Chappell et al., 2009; Groves et al., 2009). **(B)** Cerebral blood flow (CBF) quantification using pseudo-continuous ASL (pcASL) scans fitted with two-tissue compartment model in BASIL (Chappell et al., 2009; Chappell et al., 2010). This pipeline was repeated before and after acetazolamide injection.

Fitting the acquired ASE signals to the two-compartment tissue model allows us to separate OEF (our parameter of interest) and DBV per voxel. To fit our ASE signal data, we used a Bayesian framework that estimates R_2_’ and DBV, which have shown to be more separable parameters with a suitable posterior distribution for the estimation (Cherukara et al., 2019). Given the total signal $S^{Total}$ in Eq. 4, the Bayesian approach will find the optimal pair of (**DBV** and **R**_**2**_’) using a variational Bayesian (VB) inference scheme implemented in FAST ASL and BOLD Bayesian Estimation Routine (FABBER) toolkit (Woolrich et al., 2006; Chappell et al., 2009; Groves et al., 2009) in FSL (version 6.0.5, Oxford, United Kingdom), as illustrated in Figure 1A. VB inference was performed with fixed prior mean values, $\mu_{0}$ , and standard deviations, $\sigma_{0}$ The mean values for R_2_’ and DBV for healthy subjects were 2.6 s^−1^ and 3.6%, respectively (Stone and Blockley, 2017). The optimal prior standard deviation was chosen as $\sigma_{0}\left(R_{2}^{\prime }\right)=10^{\frac{3}{2}}\;s^{-1}$ and $\sigma_{0}\left(DBV\right)=10^{\frac{1}{2}}\;\%$ based on numerical simulations (Le et al., 2023). After implementing Bayesian estimation to map DBV and R_2_’ in each participant, OEF was calculated voxelwise based on the proportion between DBV and R_2_’ with known constants from Eq. 5. All qBOLD model analyses were done in native space.

#### 2.4.3 The relationship between OEF and CMRO_2_

The relationship between CMRO_2_ (μmol/100 g/min) and OEF (%) (Kety and Schmidt, 1948) can be expressed as

$$CMRO_{2}=CBF\times OEF\times {\left[Hb\right]}_{a}$$ (6)

Where CBF is the cerebral blood flow (mL/100 g/min), and ${\left[Hb\right]}_{a}$ is the oxygenated hemoglobin concentration in the arteriole (8.272 μmol/mL) This is calculated from ${\left[Hb\right]}_{a}={\left[Hb\right]}_{t}\times Y_{a}$ , where ${\left[Hb\right]}_{t}$ (8.441 μmol/mL) is the hemoglobin concentration in the tissue blood with assumption of hematocrit of *Hct* = 0.40 (Zhang et al., 2018). $Y_{a}$ is the arterial oxygenation, which is assumed to be 0.98.

#### 2.4.4 Perfusion measurements

The complete procedure to measure cerebral blood flow is shown in Figure 1B. We used the Bayesian Inference for Arterial Spin Labeling MRI (BASIL) toolkit (Chappell et al., 2009) implemented in FSL (version 6.0.5, Oxford, United Kingdom) for analyzing ASL data and quantifying perfusion maps. This approach allows fitting a two-compartment model to separate macrovascular and tissue information in ASL signal to quantify CBF maps (Groves et al., 2009; Chappell et al., 2010).

The two-compartment model assumed that the walls of arterial vessels do not allow any substance to pass through them, and that blood in the arteries moves through the voxel immediately. The total signal from any voxel is the sum of both tissue and intravascular components. CBF maps underwent a voxelwise calibration method using the measured M_0_ maps (Pinto et al., 2020). Fix label duration was applied, and initial parameter values were assumed as following: initial arterial transit time (ATT) = 1.3 s; T_1_ values for tissue (T_1t_) = 1.3 s (based on 3 T field strength); T_1_ values for blood (T_1b_) = 1.65 s; brain/blood partition coefficient (λ) = 0.9 mL/g; and labeling efficiency (α) = 0.85 (Alsop et al., 2015). Analyses to compute CBF maps were done in native ASL space.

#### 2.4.5 Image registration

To assess the regional relationship between hemodynamic parameters, especially OEF and perfusion across subjects, all quantitative maps were registered to Montreal Neurological Institute (MNI) space (Collins et al., 1999; Fonov et al., 2009; Fonov et al., 2011). For hemodynamics parameter maps, including OEF, DBV, and R_2_’, the spin echo image from the ASE scans was linearly co-registered, with FMRIB’s Linear Image Registration Tool (FLIRT) (Jenkinson and Smith, 2001; Jenkinson et al., 2002; Greve and Fischl, 2009), to subject-specific T_1_-structural space and then non-linearly co-registered, with FMRIB’s Non-linear Image Registration Tool (FNIRT) (Andersson et al., 2010), to MNI space. All parameter maps were then warped to MNI space with combined transformation parameters from those two registrations. To register CBF maps to MNI space, we first performed linear registration (FLIRT) of proton density (M_0_) images to T_1_ images; then, all CBF maps were transformed into MNI space using non-linear co-registration as described above.

### 2.5 Data analysis

#### 2.5.1 Mean ROI calculation

Before assessing parameters of interest (CBF, OEF, and R_2_’) across brain regions and calculating the average for each ROI, we excluded voxels with unphysiological extreme values. Voxels with OEF values greater than 100%, and with estimated R_2_’ greater than 20 s^−1^ were excluded from analysis. The thresholds were set consistently to prior relevant studies (Stone and Blockley, 2017; Cherukara et al., 2019). For group average parameter maps across all participants, we calculated the mean only for MNI voxels having at least 50% of the population (i.e., at least 4 subjects) within physiological values. Intermediate processing steps, including subject masks in MNI space, are shown in Supplementary Material, Supplementary Figure S1 (for OEF), Supplementary Figure S2 (for DBV), and Supplementary Figure S3 (for R_2_’).

In MNI space, the Harvard-Oxford cortical atlas (Frazier et al., 2005; Desikan et al., 2006; Makris et al., 2006; Goldstein et al., 2007) was used to delineate nine brain regions of interest (ROIs) within the DMN. Regions within the DMN included the posterior cingulate gyrus (pCG), precuneus (PCun), anterior cingulate gyrus (aCG), angular gyrus (AG), supramarginal gyrus (SG) (including in inferior parietal lobe), and medial frontal gyrus (MFG) (Broyd et al., 2009). As a reference for comparison for this study, we chose other cortical brain regions, including the occipital pole (OP), inferior temporal gyrus (ITG), and middle temporal gyrus (MTG), to represent non-DMN regions.

#### 2.5.2 Statistical analysis

All statistical analyses were performed using MATLAB R2021a (MathWorks, Natick, MA, 2016). Differences between before and after vasodilation were assessed using a pairwise two-way *t*-test with Bonferroni correction for nine ROIs. A *p*-value of less than 0.006 (where $\alpha =\frac{0.05}{number\;of\;comparisons}$ ) was considered statistically significant for this analysis.

To evaluate the relationship of OEF and CBF across regions during vasodilation, a linear mixed-effects model was conducted using OEF as the dependent variable and CBF as the independent variable, with subject and region as random effects. We also investigated the correlation of R_2_’ and CBF across regions during vasodilation, using a separate linear mixed-effects model with R_2_’ as the dependent variable and CBF as the independent variable. For both models, subjects and regions were used as random effects to adjust for inherent physiological variations across individuals and regions. Each mixed model was fit separately using the MATLAB software package *“fitlme”* (Pinheiro and Bates, 1996). A *p*-value of less than 0.05 was considered statistically significant.

## 3 Results

**FIGURE 2:**
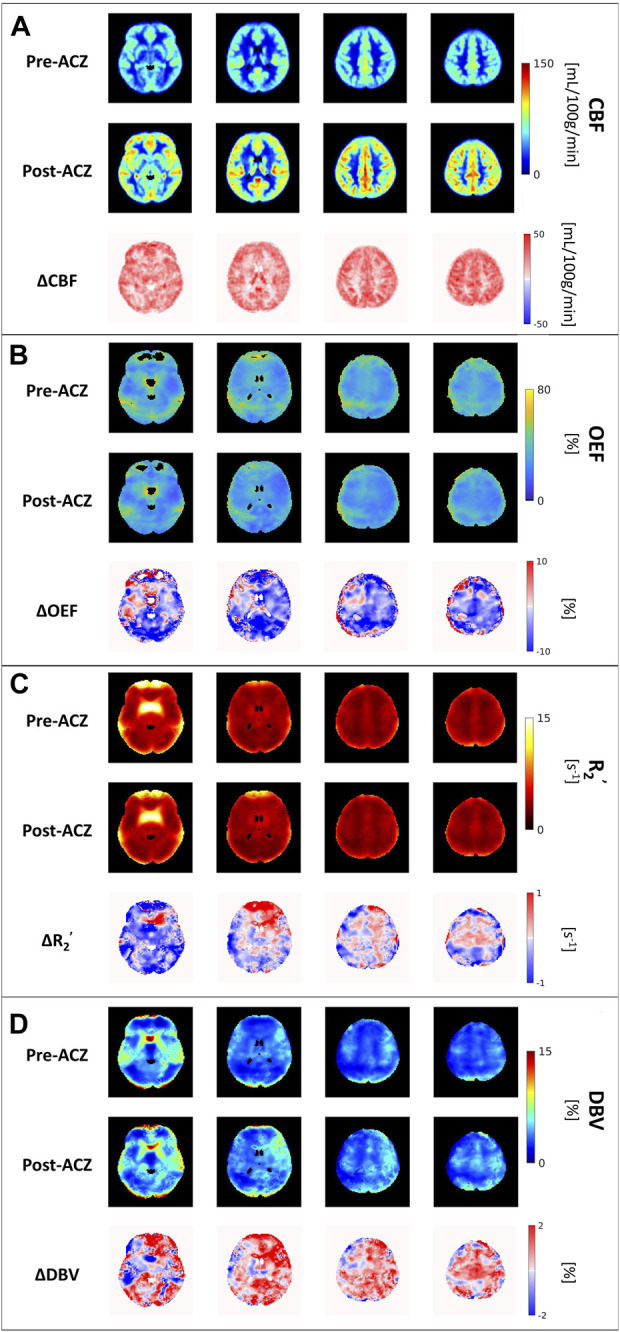
The group average of **(A)** cerebral blood flow (CBF) maps (in mL/100 g/min), **(B)** oxygen extraction fraction (OEF) maps (in %), **(C)** transverse relaxation rate (R_2_^’^) (in s^−1^) and **(D)** deoxygenated blood volume (DBV) across all eight healthy subjects before (Pre-ACZ) and after (Post-ACZ) vasodilation with the corresponding absolute difference maps between two conditions for each parameter (ΔCBF, ΔOEF, ΔR_2_^’^, and ΔDBV). All maps were registered to Montreal Neurological Institute (MNI) space.

All eight healthy participants received MRI scans consisting of the GESEPI-ASE sequence for quantitative BOLD modeling to estimate hemodynamic parameters, including R_2_’, DBV, and OEF (our parameter of interest). After excluding unphysiological voxels, group average maps of each parameter before and after vasodilation are shown in Figure 2. Excluded voxels were primarily located in frontal brain areas prone to air-tissue bulk magnetic susceptibility effects. The percentage of voxels removed due to thresholding in the two conditions is shown in Supplementary Table S1 (Pre-ACZ: DBV = 0.21%, R_2_’ = 2.05%, OEF = 13.1%; Post-ACZ: DBV = 0.16%, R_2_’ = 1.84%, OEF = 10.93%. We focused on parameters that are sensitive to brain oxygenation (OEF and R_2_’) and perfusion; the corresponding DBV maps are shown in Figure 2.

**FIGURE 3:**
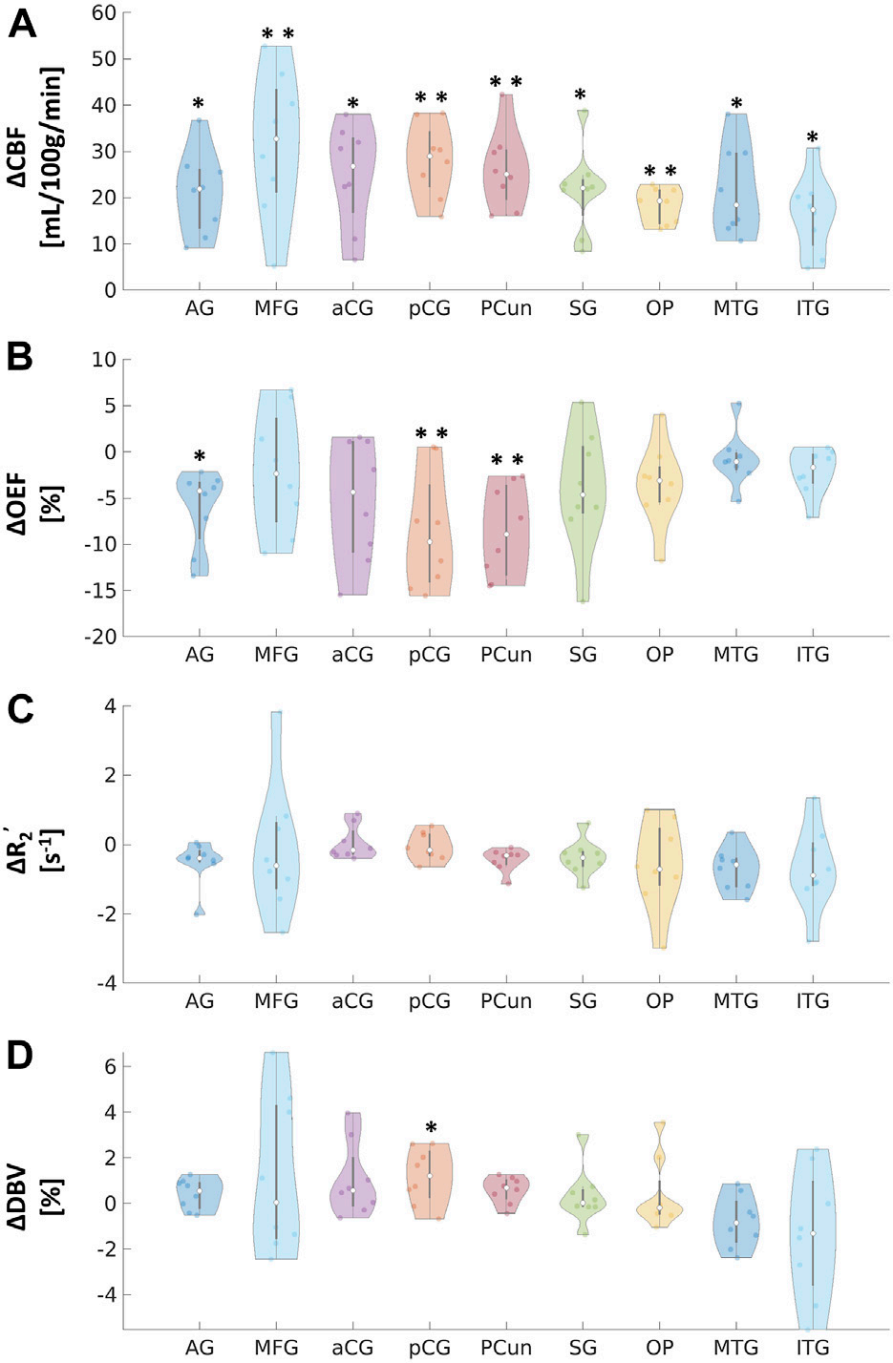
Absolute changes in **(A)** cerebral blood flow (CBF) in mL/100 g/min, **(B)** oxygen extraction fraction (OEF) in %, **(C)** transverse relaxation rate (R_2_^’^) in s^−1^, and **(D)** deoxygenated blood volume (DBV) in % in response to acetazolamide (ACZ) in different regions of interest (ROIs): angular gyrus (AG), medial frontal gyrus (MFG), anterior cingulate gyrus (aCG), posterior cingulate gyrus (pCG), precuneus (Pcun), occipital pole (OP), supramarginal gyrus (SG), middle temporal gyrus (MTG), and inferior temporal gyrus (ITG). All tests were performed using pairwise two-way *t*-tests between pre- and post-vasodilation with Bonferroni correction. (*p < 0.05, significant raw *p*-value; **p < 0.006, significant *p*-value after correction).

Compared to the baseline condition, increased CBF and decreased OEF were observed across multiple areas in the brain at the group average level (Figures 2A, B). We also observed a decreasing trend in R_2_’ (Figure 2C) and an increasing trend in DBV (Figure 2D) during vasodilation. Mean parameter values, including CBF, OEF, R_2_’, DBV and CMRO_2_ for all subjects across selected regions for each condition are presented in Table 1. Additionally, the distribution of changes in OEF, R_2_’, DBV and CBF in response to ACZ for each selected region were plotted in Figure 3.

**TABLE 1:** Average cerebral blood flow (CBF) (in mL/100 g/min), oxygen extraction fraction (OEF) (in %), transverse relaxation rate (R_2_^’^) (in s^−1^), deoxygenated blood volume (DBV) (in %) and cerebral metabolic rate of oxygen (CMRO_2_) (in μmol/100 g/min) before (Pre) and after (Post) vasodilation across all healthy subjects (mean ± std) in different regions of interest (ROIs): angular gyrus (AG), medial frontal gyrus (MFG), anterior cingulate gyrus (aCG), posterior cingulate gyrus (pCG), precuneus (PCun), occipital pole (OP), supramarginal gyrus (SG), middle temporal gyrus (MTG), and inferior temporal gyrus (ITG). All tests were performed using a two-sided paired *t*-test with Bonferroni correction. (*p < 0.05, significant raw *p*-value; **p < 0.006, significant *p*-value after correction).

		AG	MFG	aCG	pCG	PCun	SG	OP	MTG	ITG
CBF (mL/100 g/min)	Pre-ACZ	60.7 ± 15.6	43.2 ± 10.6	68.6 ± 14.5	76.3 ± 14.8	61.6 ± 11.1	54.9 ± 14.1	40.0 ± 9.7	54.5 ± 13.7	40.2 ± 10.7
	Post-ACZ	81.8 ± 22.4	74.8 ± 23.2	93.3 ± 21.3	104.4 ± 17.8	87.6 ± 18.1	76.4 ± 22.2	58.4 ± 8.8	76.1 ± 20.7	57.6 ± 13.5
	P-val	0.046*	0.004**	0.017*	0.004**	0.004**	0.037*	0.001**	0.027*	0.018*
OEF (%)	Pre-ACZ	44.4 ± 4.1	32.4 ± 10.9	35.9 ± 4.7	34.5 ± 4.8	39.6 ± 4.2	43.2 ± 5.2	30.7 ± 8.3	25.9 ± 3.0	29.3 ± 3.1
	Post-ACZ	38.2 ± 3.8	30.3 ± 11.6	30.7 ± 6.5	25.7 ± 4.7	30.9 ± 4.1	39.1 ± 2.5	27.2 ± 7.2	25.1 ± 3.0	27.1 ± 2.3
	P-val	0.008*	0.717	0.085	0.003**	0.001**	0.070	0.381	0.609	0.134
R_2_^’^ (s^−1^)	Pre-ACZ	4.8 ± 1.0	9.1 ± 2.6	3.8 ± 0.4	3.7 ± 0.3	4.0 ± 0.7	5.1 ± 0.5	7.1 ± 1.6	7.8 ± 1.0	11.1 ± 1.2
	Post-ACZ	4.3 ± 0.8	9.0 ± 2.1	3.9 ± 0.7	3.7 ± 0.5	3.6 ± 0.7	4.7 ± 0.6	6.5 ± 0.7	7.1 ± 0.8	10.4 ± 1.9
	P-val	0.270	0.900	0.856	0.816	0.233	0.199	0.348	0.154	0.404
DBV (%)	Pre-ACZ	3.1 ± 0.7	11.0 ± 6.1	3.2 ± 0.4	3.4 ± 0.8	3.2 ± 0.7	3.1 ± 0.9	7.8 ± 2.2	11.5 ± 2.8	17.6 ± 3.1
	Post-ACZ	3.5 ± 0.7	12.2 ± 6.0	4.3 ± 1.4	4.5 ± 1.3	3.7 ± 0.7	3.4 ± 0.9	8.2 ± 2.1	10.7 ± 2.5	16.1 ± 3.6
	P-val	0.270	0.694	0.067	0.045*	0.114	0.496	0.721	0.550	0.425
CMRO_2_ (μmol/100 g/min)	Pre-ACZ	223.0 ± 58.2	117.1 ± 47.7	206.3 ± 57.1	216.9 ± 51.8	201.7 ± 43.8	194.9 ± 51.3	103.2 ± 38.9	115.6 ± 28.8	100.3 ± 28.1
	Post-ACZ	260.8 ± 80.1	173.5 ± 40.6	238.4 ± 82.3	221.5 ± 51.1	223.1 ± 54.0	246.7 ± 70.6	130.4 ± 35.8	154.9 ± 33.5	130.3 ± 35.8
	P-val	0.298	0.023*	0.379	0.859	0.399	0.116	0.168	0.025*	0.083

Paired two-way *t*-test with Bonferroni correction showed statistically significant differences in CBF and OEF estimates in selected regions during vasodilation (i.e., Pre-ACZ versus Post-ACZ), as presented in Table 1. With the injection of ACZ, CBF increased across all chosen regions before correction for multiple comparisons. After correction, elevated CBF remained significant in MFG ( P=0.004 ), pCG ( P=0.004 ), PCun ( P=0.004 ), and OP ( P=0.001 ) regions. In parallel, a significant reduction in OEF from qBOLD was observed only in pCG ( P=0.003 ) and PCun ( P=0.001 ) after correction, with a trend of decreased OEF also in the angular gyrus. In addition, a slight trend of DBV increase during vasodilation was observed only in pCG ( P=0.045 ) before correction. For R_2_’, there was a small reduction trend during vasodilation; however, we found no region showing statistically significant change with ACZ (Table 1). Additionally, while there was a trend of increased CMRO_2_ after vasodilation in several brain areas, especially the MFG and MTG, CMRO_2_ showed no significant change in all regions after correction for multiple comparisons (Table 1).

**FIGURE 4:**
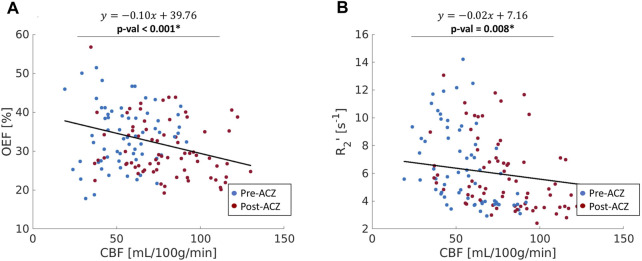
**(A)** The correlation between oxygen extraction fraction OEF (%) and cerebral blood flow (CBF); **(B)** the relationship between R_2_^’^ (s^−1^) and CBF across all healthy subjects in nine ROIs (red: before vasodilation (Pre-ACZ); blue: after vasodilation (Post-ACZ). The solid black line represents the fitted line from mixed-effect models. Linear mixed-effect models with adjustment for subject clustering and region were performed separately for OEF and R_2_^’^. *p* < 0.05* Mixed-effects model: OEF∼CBF + (1|Region) + (1|Subject).

The CBF response to ACZ showed a significant negative linear relationship to OEF value at baseline and after vasodilation using a mixed-linear effects model with subject clustering ( β=−0.104±0.027 ; t=−3.852,P<0.001 ) (Figure 4A; Table 2). We further investigated the relationship between CBF and R_2_’ using the same mixed-effects model. The CBF response to ACZ was also correlated with R_2_’ ( β=−0.016±0.006 ; t=−2.692,P=0.008 ) (Figure 4B; Table 2).

**TABLE 2:** Linear mixed-effects models between OEF, R_2_^’^, and CBF in the whole group (*N* = 8) across 9 ROIs. In both models, subjects and regions were used as random effects. *p < 0.05 β : standardized beta coefficient; SE: standardized error; OEF: oxygen extraction fraction; R_2_’: transverse relaxation rate; CBF: cerebral blood flow.

Dependent variables	Independent variables	β±SE	*t*-statistics	*p*-value
OEF	CBF	−0.104 ± 0.027	−3.852	<0.001*
R_2_^’^	CBF	−0.016 ± 0.006	−2.692	0.008*

## 4 Discussion

The purpose of this study was to demonstrate qBOLD modeling of ASE scans in healthy volunteers to detect regional OEF decreases in key ROIs of the DMN during concomitant perfusion increase with ACZ vasodilation. The main findings were as follows: 1) Across baseline and vasodilation states, OEF was inversely related to quantitative perfusion as expected, indicating a compensation to maintain oxygen metabolism and reliability of the local OEF measures. 2) Similar changes with ACZ observed using R_2_’ relaxation estimates from the same ASE acquisitions, which highlights the utility of qBOLD compartment modeling to non-invasively measure specific OEF imaging markers. 3) OEF reduction was observed in critical DMN regions, including the precuneus and posterior cingulate gyrus.

### 4.1 Baseline OEF and change with acetazolamide in healthy participants

The observed mean baseline OEF across selected brain regions of 35.1% is consistent with the physiological range of previous PET and MRI studies (Yamaguchi et al., 1986; Leenders et al., 1990; Cho et al., 2021; Jiang et al., 2023). We observed an OEF reduction of 6.2%–8.7% (absolute oxygenation) in significant DMN regions, which was smaller in magnitude than the 15.6% absolute OEF decrease in the sagittal sinus from past ACZ studies on healthy controls (Václavů et al., 2020). This discrepancy could be due to methodological differences, as previous studies focused on global OEF values and used a distinct contrast mechanism and calibration with T_2_-prepared inversion recovery sequences (Václavů et al., 2020; Baas et al., 2022). The qBOLD method adopted here may also have lower sensitivity to OEF changes due to noise contributions in the model fitting *in vivo*. Of note, the T_2_-based study also reported a larger magnitude of perfusion increase (69.3% increase) using pcASL with ACZ than our study (average 43.4% increase across regions). Therefore, direct comparison of the OEF imaging approaches in the same participants and improvement to sensitivity of our qBOLD approach is warranted in future work.

### 4.2 Correlation between hemodynamic parameters and perfusion

Although R_2_’ has been used as a surrogate marker for brain OEF in previous studies (Ni et al., 2015), including identification of putative oxygenation changes in cerebrovascular disease (Ni et al., 2017; Kaczmarz et al., 2021), this work suggests that qBOLD compartmental modeling outperforms R_2_’ measures in detecting physiological OEF changes in healthy volunteers. While significant OEF reductions were measured in the pCG and PCun with qBOLD, none of the ROIs showed a significant R_2_^’^ change with ACZ. Additionally, the expected inverse relationship between OEF and CBF across baseline and vasodilatory states was stronger using qBOLD than between R_2_’ and CBF. One rationale could be that R_2_’ is proportional to both OEF and DBV, such that OEF reductions concurrently with potential DBV elevations during vasodilation lead to overall smaller changes in R_2_’ values. Because qBOLD compartment modeling uses multiple asymmetric echoes to disentangle contributions from OEF and DBV, the resulting regional OEF measures are likely to be more sensitive than R_2_’, as observed in our results. In addition to the Bayesian fitting for R_2_’, a supplementary analysis of the monoexponential fits based on streamline qBOLD (Stone and Blockley, 2017) was also performed. Our more complex modeling approach may produce R_2_’ measures that are noisier compared to monoexponential fits for the relaxation parameter (Supplementary Figure S5), which may contribute to lack of sensitivity of R_2_’ to changes during vasodilation. Besides, in whole group level analysis with monoexponential fits, there was a slight decrease for R_2_’ in pCG $\left(P=0.028\right)$ and OP $\left(P=0.039\right)$ before correction (Supplementary Table S2). However, in this analysis, we chose to compare OEF and R_2_’ values that were both derived from Bayesian fitting for consistency. R_2_’ measures are also susceptible to contributions from orientation effects of the blood vessels (Kaczmarz et al., 2021) and artifacts from non-heme susceptibility sources (iron, myelin). These contributions will also manifest differently on OEF values from qBOLD modeling, which requires further evaluation with related multi-parametric qBOLD approaches that acquire separate R_2_ and R_2_* maps measures to assess oxygenation (Gersing et al., 2015). Such multi-parametric qBOLD have been used to identify reduced baseline oxygen metabolism in the affected middle cerebral artery territory with moderate ischemia and may provide an alternative to ASE acquisitions (Bouvier et al., 2015).

A good validation for our qBOLD method to measure OEF is to test the hypothesis that CMRO_2_ is unchanged during vasodilation, i.e., that OEF decreases are commensurate with the increase in CBF during acetazolamide. From this study, we found no significant CMRO_2_ change after Bonferroni correction in all selected regions, which is consistent with our hypothesis and reflects reliability of the qBOLD method. However, we observed a slight increasing trend in CMRO_2_, which may reflect residual underestimation bias in OEF values (i.e., underestimating the OEF decrease) and limited sensitivity of the qBOLD model to OEF. While CBF significantly increased in all selected regions, OEF only showed a significant decrease in pCG and PCun (Table 1). Therefore, there was an overall trend of increased CMRO_2_ in several regions, even though we observed the expected compensation of OEF and CBF during vasodilation. Future work will enhance the accuracy and sensitivity of qBOLD modeling with refined physiological prior information and improved fitting routines that are more robust to noise contributions.

### 4.3 Effects of vasodilation in cerebral regions

This study imaged the regional effects of vasodilation on OEF across the entire cerebral cortex, focusing on DMN (Figures 2, 3). We found that most DMN regions showed increased CBF during vasodilation, and decreased OEF was observed in pCG and PCun. In contrast, the non-DMN regions did not show a significant response in any hemodynamic parameters. One potential implication of this finding is that DMN regions show a strong sensitivity to vasodilation in those regions, which is consistent with well-established mechanisms of vasodilation with ACZ (Kleinschmidt et al., 1995; Wang et al., 2015). In addition, BOLD fMRI studies during hypercapnia have identified co-fluctuations in brain hemodynamics that spatially resemble the DMN but are responsive to vasoactive (not neuronal) stimuli (Bright et al., 2020). Such “vascular networks” may have unique OEF properties at baseline and in vasodilated conditions, and methods such as the qBOLD approach can characterize such physiological mechanisms of brain functional network emergence. These capabilities will enable important future studies to assess the link between oxygen metabolism in other brain networks and their disorders. Outside of the DMN, the medial temporal lobe (MTL) has been known as a key early region that is affected by many brain diseases (Clifford R. Jack et al., 1997; Tam et al., 2005; Burton et al., 2009). OEF can serve as a functional biomarker due to its sensitivity in the early stages of brain diseases and aging, as neural activity is tightly linked to the brain’s oxygen consumption (Watts et al., 2018). At baseline, average OEF values in the medial temporal lobe were measured to be 25.9% ± 3.0% (Table 1), consistent with prior MRI studies’ measurement of OEF in vessels supplying the MTL of 23.9% using T_2_-relaxation-under-phase-contrast approach (Jiang et al., 2023). Even though there was a decreasing trend, we observed no significant change in MTL OEF during vasodilation ( $\left.P=0.609\right)$ . This observation contrasts with Jiang et al., who observed increased MTL OEF after vasoactive challenge with caffeine ingestion. Caffeine is a common vasoconstrictor, which is known to reduce CBF and increase OEF to maintain the same amount of total oxygen consumption (Xu et al., 2015). Both ACZ and caffeine are vasoactive stimuli with minimal neuroactive effects, although they are in opposite directions. For the MTL, Jiang et al. observed a 9.1% OEF increase due to caffeine, while our study observed an average of 12.7% OEF decrease across regions due to ACZ vasodilation. More studies are needed to characterize the accuracy of qBOLD to detect changes in MTL OEF with physiological changes or aging and may require additional corrections for signal loss or distortion due to susceptibility effects in some MTL subregions (Olman et al., 2009).

### 4.4 Limitations

To perform qBOLD modeling, we have used an ASE sequence to directly estimate R_2_’ with consistent R_2_-weighting across echoes (An and Lin, 2003). One main limitation of the qBOLD model is that the separation of DBV and OEF effects in the qBOLD model depends mainly on the subtle change in decay patterns. Therefore, it requires high signal-to-noise-ratio (SNR) to accurately estimate OEF from the ASE signals (An and Lin, 2000). Due to challenging low SNR from our sequence, OEF values estimated from qBOLD model still had unreasonable physiological values in particular voxels that we had to remove. To address this noise limitation, a benefit to fitting this multi-compartment with the Bayesian framework is the ability to include prior knowledge to improve parameter estimates. In this study, prior mean values and standard deviations were taken from previous studies (Stone and Blockley, 2017; Cherukara et al., 2019; Le et al., 2023). Moreover, it has been suggested that prior means do not significantly affect the parameter estimation (Cherukara et al., 2019). However, the prior standard deviations were optimized based on a broader range of R_2_’-weighted images (24 R_2_’-images in Cherukara et al., 2019, and 14 in Le et al., 2023) than acquired in this MRI protocol. For this data, we also investigated the effect of prior standard deviations to the parameter estimation by comparing between the chosen prior standard deviations ( $\left.{\sigma \left(DBV\right)=10}^{\frac{3}{2}}\;\%;\;{\sigma \left(R_{2}^{\prime }\right)=10}^{\frac{1}{2}}\;s^{-1}\right)$ with a broader one ( $\left.{\sigma \left(DBV\right)=10}^{\frac{5}{2}}\;\%;\;{\sigma \left(R_{2}^{\prime }\right)=10}^{\frac{5}{2}}s^{-1}\right)$ . We found that the broader standard deviation, which places less weight on prior information, resulted in more unphysiological values compared to our chosen value as shown in Supplementary Table S1. Further optimization for prior information will be assessed to maintain a robust estimation of OEF.

*Hct* was assumed as 0.40 based on the literature value for general circulation (Nicoll et al., 2012), which is consistent with average hematocrit in healthy subjects from other studies (Brown et al., 1962; Jeong et al., 2021). On the other hand, prior relevant studies have used a lower *Hct* of 0.34 (He and Yablonskiy, 2007) because of lower *Hct* in small vessels. Because *Hct* influences quantification specifically of the intravascular compartment, for our two-compartment model, changing *Hct* has a non-linear effect on the parameter estimates. For instance, using a *Hct* of 0.34 (i.e., a 15% lower value of *Hct*) and without spatial regularization, Cherakura et al. observed the same mean DBV values but a 3% higher mean OEF values compared to analysis with *Hct* of 0.40. However, it is unclear how much the *Hct* varies throughout the brain, which may propagate to additional regional differences of OEF estimates from the qBOLD signal. Finally, this study adopted a Bayesian framework that aims for the maximal free energy of the approximate posterior to better estimate the data (Chappell et al., 2009). This approach was chosen to ensure that applying additional smoothing (Gaussian smoothing with kernel of 4 mm) did not affect the fitting. Because the qBOLD-OEF measurement requires more complex models, it is more sensitive to the presence of noise compared to CBF quantification from ASL. Therefore, we applied a larger kernel of smoothing (i.e., 4 mm) for OEF, which is consistent to previous studies (Cho et al., 2021; Uchida et al., 2022). This kernel was empirically chosen after comparing multiple kernel sizes on our data to reduce unstructured noise and heterogeneity in the OEF maps but has the drawback of decreasing the spatial resolution of regional OEF measures. The relatively small sample size of our study is also a limitation and should be expanded in future studies to include larger cohorts with various ages and cerebrovascular conditions.

## 5 Conclusion

We demonstrated that regional OEF measurements from qBOLD are reliable across multiple physiological conditions and evaluated OEF through correlation with ASL perfusion in response to ACZ vasodilation as reliable indicators of tissue health in healthy cohort. This study enhances our understanding of the relationship between OEF and CBF with regional alterations in brain vasculature and has implications for the future use of oxygenation MRI to study vascular alterations in cognitive impairment and cerebrovascular diseases.

## Data Availability

The datasets presented in this study can be found in online repositories. The names of the repository/repositories and accession number(s) can be found below: https://github.com/fanlab-ucdavis/hemodynamics-data.

## References

[B1] Alsop D. C. Detre J. A. Golay X. Günther M. Hendrikse J. Hernandez-Garcia L. (2015). Recommended implementation of arterial spin-labeled perfusion MRI for clinical applications: A consensus of the ismrm perfusion study group and the European consortium for ASL in dementia. Magn. Reson. Med. 73, 102–116. 10.1002/mrm.25197 24715426 PMC4190138

[B2] An H. Lin W. (2003). Impact of intravascular signal on quantitative measures of cerebral oxygen extraction and blood volume under normo- and hypercapnic conditions using an asymmetric spin echo approach. Magn. Reson. Med. 50, 708–716. 10.1002/mrm.10576 14523956

[B3] An H. Lin W. (2000). Quantitative measurements of cerebral blood oxygen saturation using magnetic resonance imaging. J. Cereb. Blood Flow. Metab. 20, 1225–1236. 10.1097/00004647-200008000-00008 10950383 PMC4096835

[B4] Andersson J. L. R. Jenkinson M. Smith S. (2010). Non-linear registration, aka spatial normalisation FMRIB technical report TR07JA2.

[B5] Baas K. P. A. Coolen B. F. Petersen E. T. Biemond B. J. Strijkers G. J. Nederveen A. J. (2022). Comparative analysis of blood T2 values measured by T2 -trir and TRUST. J. Magn. Reson Imaging 56, 516–526. 10.1002/jmri.28066 35077595

[B6] Blockley N. P. Stone A. J. (2016). Improving the specificity of R2’ to the deoxyhaemoglobin content of brain tissue: prospective correction of macroscopic magnetic field gradients. NeuroImage 135, 253–260. 10.1016/j.neuroimage.2016.04.013 27150229

[B7] Bouvier J. Detante O. Tahon F. Attye A. Perret T. Chechin D. (2015). Reduced CMRO₂ and cerebrovascular reserve in patients with severe intracranial arterial stenosis: A combined multiparametric qBOLD oxygenation and BOLD fMRI study. Hum. Brain Mapp. 36, 695–706. 10.1002/hbm.22657 25307948 PMC6869377

[B8] Bright M. G. Whittaker J. R. Driver I. D. Murphy K. (2020). Vascular physiology drives functional brain networks. NeuroImage 217, 116907. 10.1016/j.neuroimage.2020.116907 32387624 PMC7339138

[B9] Brown E. Hopper J. Hodges J. L. Bradley B. Wennesland R. Yamauchi H. (1962). Red cell, plasma, and blood volume in the healthy women measured by radiochromium cell-labeling and hematocrit. J. Clin. Invest. 41, 2182–2190. 10.1172/JCI104677 14015940 PMC291153

[B10] Brown W. R. Thore C. R. (2011). Review: cerebral microvascular pathology in ageing and neurodegeneration. Neuropathol. Appl. Neurobiol. 37, 56–74. 10.1111/j.1365-2990.2010.01139.x 20946471 PMC3020267

[B11] Broyd S. J. Demanuele C. Debener S. Helps S. K. James C. J. Sonuga-Barke E. J. S. (2009). Default-mode brain dysfunction in mental disorders: A systematic review. Neurosci. Biobehav. Rev. 33, 279–296. 10.1016/j.neubiorev.2008.09.002 18824195

[B12] Buch S. Ye Y. Haacke E. M. (2017). Quantifying the changes in oxygen extraction fraction and cerebral activity caused by caffeine and acetazolamide. J. Cereb. Blood Flow. Metab. 37, 825–836. 10.1177/0271678X16641129 27029391 PMC5363462

[B13] Burton E. J. Barber R. Mukaetova-Ladinska E. B. Robson J. Perry R. H. Jaros E. (2009). Medial temporal lobe atrophy on MRI differentiates alzheimer’s disease from dementia with lewy bodies and vascular cognitive impairment: A prospective study with pathological verification of diagnosis. Brain 132, 195–203. 10.1093/brain/awn298 19022858

[B14] Chappell M. A. Groves A. R. Whitcher B. Woolrich M. W. (2009). Variational bayesian inference for a nonlinear forward model. IEEE Trans. Signal Process. 57, 223–236. 10.1109/TSP.2008.2005752

[B15] Chappell M. A. MacIntosh B. J. Donahue M. J. Günther M. Jezzard P. Woolrich M. W. (2010). Separation of macrovascular signal in multi-inversion time arterial spin labelling MRI. Magn. Reson. Med. 63, 1357–1365. 10.1002/mrm.22320 20432306

[B16] Cherukara M. T. Stone A. J. Chappell M. A. Blockley N. P. (2019). Model-based Bayesian inference of brain oxygenation using quantitative BOLD. NeuroImage 202, 116106. 10.1016/j.neuroimage.2019.116106 31430532 PMC7334042

[B17] Cho J. Lee J. An H. Goyal M. S. Su Y. Wang Y. (2021). Cerebral oxygen extraction fraction (OEF): comparison of challenge-free gradient echo qsm+qbold (qq) with 15o pet in healthy adults. J. Cereb. Blood Flow. Metab. 41, 1658–1668. 10.1177/0271678X20973951 33243071 PMC8221765

[B18] Cleland N. R. W. Al-Juboori S. I. Dobrinskikh E. Bruce K. D. (2021). Altered substrate metabolism in neurodegenerative disease: new insights from metabolic imaging. J. Neuroinflammation 18, 248. 10.1186/s12974-021-02305-w 34711251 PMC8555332

[B19] Clifford R. Jack J. Petersen R. C. Yue Cheng Xu Waring S. C. O’Brien P. C. (1997). Medial temporal atrophy on MRI in normal aging and very mild Alzheimer’s disease. Neurology 49, 786–794. 10.1212/WNL.49.3.786 9305341 PMC2730601

[B20] Coalson T. S. Van Essen D. C. Glasser M. F. (2018). The impact of traditional neuroimaging methods on the spatial localization of cortical areas. Proc. Natl. Acad. Sci. 115, E6356–E6365. 10.1073/pnas.1801582115 29925602 PMC6142239

[B21] Collins D. L. Zijdenbos A. P. Baaré W. F. C. Evans A. C. (1999). “ANIMAL+INSECT: improved cortical structure segmentation,” in Information processing in medical imaging lecture notes in computer science. Kuba A. Šáamal M. Todd-Pokropek A. (Berlin, Heidelberg: Springer), 210–223. 10.1007/3-540-48714-X_16

[B22] Desikan R. S. Ségonne F. Fischl B. Quinn B. T. Dickerson B. C. Blacker D. (2006). An automated labeling system for subdividing the human cerebral cortex on MRI scans into gyral based regions of interest. NeuroImage 31, 968–980. 10.1016/j.neuroimage.2006.01.021 16530430

[B23] Fan A. P. Khalil A. A. Fiebach J. B. Zaharchuk G. Villringer A. Villringer K. (2020). Elevated brain oxygen extraction fraction measured by MRI susceptibility relates to perfusion status in acute ischemic stroke. J. Cereb. Blood Flow. Metab. 40, 539–551. 10.1177/0271678X19827944 30732551 PMC7026852

[B24] Fan J.-L. Nogueira R. C. Brassard P. Rickards C. A. Page M. Nasr N. (2022). Integrative physiological assessment of cerebral hemodynamics and metabolism in acute ischemic stroke. J. Cereb. Blood Flow. Metab. Off. J. Int. Soc. Cereb. Blood Flow. Metab. 42, 454–470. 10.1177/0271678X211033732 PMC8985442 34304623

[B25] Fonov V. Evans A. C. Botteron K. Almli C. R. McKinstry R. C. Collins D. L. (2011). Unbiased average age-appropriate atlases for pediatric studies. NeuroImage 54, 313–327. 10.1016/j.neuroimage.2010.07.033 20656036 PMC2962759

[B26] Fonov V. Evans A. McKinstry R. Almli C. Collins D. (2009). Unbiased nonlinear average age-appropriate brain templates from birth to adulthood. NeuroImage 47, S102. 10.1016/S1053-8119(09)70884-5

[B27] Frazier J. A. Chiu S. Breeze J. L. Makris N. Lange N. Kennedy D. N. (2005). Structural brain magnetic resonance imaging of limbic and thalamic volumes in pediatric bipolar disorder. Am. J. Psychiatry 162, 1256–1265. 10.1176/appi.ajp.162.7.1256 15994707

[B28] Groves A. R. Chappell M. A. Woolrich M. W. (2009). Combined spatial and non-spatial prior for inference on MRI time-series. NeuroImage 45, 795–809. 10.1016/j.neuroimage.2008.12.027 19162204

[B29] Gersing A. S. Ankenbrank M. Schwaiger B. J. Toth V. Janssen I. Kooijman H. (2015). Mapping of cerebral metabolic rate of oxygen using dynamic susceptibility contrast and blood oxygen level dependent MR imaging in acute ischemic stroke. Neuroradiology 57, 1253–1261. 10.1007/s00234-015-1592-7 26364182

[B30] Goldstein J. M. Seidman L. J. Makris N. Ahern T. O’Brien L. M. Caviness V. S. (2007). Hypothalamic abnormalities in schizophrenia: sex effects and genetic vulnerability. Biol. Psychiatry 61, 935–945. 10.1016/j.biopsych.2006.06.027 17046727

[B31] Greve D. N. Fischl B. (2009). Accurate and robust brain image alignment using boundary-based registration. NeuroImage 48, 63–72. 10.1016/j.neuroimage.2009.06.060 19573611 PMC2733527

[B32] Guilliams K. P. Fields M. E. Ragan D. K. Eldeniz C. Binkley M. M. Chen Y. (2018). Red cell exchange transfusions lower cerebral blood flow and oxygen extraction fraction in pediatric sickle cell anemia. Blood 131, 1012–1021. 10.1182/blood-2017-06-789842 29255068 PMC5833262

[B33] Gupta A. Baradaran H. Schweitzer A. D. Kamel H. Pandya A. Delgado D. (2014). Oxygen extraction fraction and stroke risk in patients with carotid stenosis or occlusion: A systematic review and meta-analysis. Am. J. Neuroradiol. 35, 250–255. 10.3174/ajnr.A3668 23945227 PMC7965748

[B34] Haight T. J. Bryan R. N. Erus G. Davatzikos C. Jacobs D. R. D’Esposito M. (2015). Vascular risk factors, cerebrovascular reactivity, and the default-mode brain network. NeuroImage 115, 7–16. 10.1016/j.neuroimage.2015.04.039 25917517 PMC4469180

[B35] Handwerker D. A. Gazzaley A. Inglis B. A. D’Esposito M. (2007). Reducing vascular variability of fMRI data across aging populations using a breathholding task. Hum. Brain Mapp. 28, 846–859. 10.1002/hbm.20307 17094119 PMC6871393

[B36] He X. Yablonskiy D. A. (2007). Quantitative BOLD: mapping of human cerebral deoxygenated blood volume and oxygen extraction fraction: default state. Magn. Reson. Med. 57, 115–126. 10.1002/mrm.21108 17191227 PMC3971521

[B37] Hokari M. Kuroda S. Shiga T. Nakayama N. Tamaki N. Iwasaki Y. (2008). Combination of a mean transit time measurement with an acetazolamide test increases predictive power to identify elevated oxygen extraction fraction in occlusive carotid artery diseases. J. Nucl. Med. 49, 1922–1927. 10.2967/jnumed.108.054379 18997043

[B38] Imaizumi M. Kitagawa K. Oku N. Hashikawa K. Takasawa M. Yosmkawa T. (2004). Clinical significance of cerebrovascular reserve in acetazolamide challenge —Comparison with acetazolamide challenge H2O-PET and gas-PET. Ann. Nucl. Med. 18, 369–374. 10.1007/BF02984479 15462398

[B39] Ishii K. Kitagaki H. Kono M. Mori E. (1996). Decreased medial temporal oxygen metabolism in alzheimer’s disease shown by PET. J. Nucl. Med. 37, 1159–1165. 8965188

[B40] Jenkinson M. Bannister P. Brady M. Smith S. (2002). Improved optimization for the robust and accurate linear registration and motion correction of brain images. NeuroImage 17, 825–841. 10.1016/s1053-8119(02)91132-8 12377157

[B41] Jenkinson M. Beckmann C. F. Behrens T. E. J. Woolrich M. W. Smith S. M. (2012). Fsl. NeuroImage 62, 782–790. 10.1016/j.neuroimage.2011.09.015 21979382

[B42] Jenkinson M. Smith S. (2001). A global optimisation method for robust affine registration of brain images. Med. Image Anal. 5, 143–156. 10.1016/S1361-8415(01)00036-6 11516708

[B43] Jeong H. R. Shim Y. S. Lee H. S. Hwang J. S. (2021). Hemoglobin and hematocrit levels are positively associated with blood pressure in children and adolescents 10 to 18 years old. Sci. Rep. 11, 19052. 10.1038/s41598-021-98472-0 34561491 PMC8463603

[B44] Jiang D. Lin Z. Liu P. Sur S. Xu C. Hazel K. (2020). Brain oxygen extraction is differentially altered by alzheimer’s and vascular diseases. J. Magnetic Reson. Imaging 52, 1829–1837. 10.1002/jmri.27264 PMC9973301 32567195

[B45] Jiang D. Liu P. Lin Z. Hazel K. Pottanat G. Lucke E. (2023). MRI assessment of cerebral oxygen extraction fraction in the medial temporal lobe. NeuroImage 266, 119829. 10.1016/j.neuroimage.2022.119829 36565971 PMC9878351

[B46] Kaczmarz S. Göttler J. Petr J. Hansen M. B. Mouridsen K. Zimmer C. (2021). Hemodynamic impairments within individual watershed areas in asymptomatic carotid artery stenosis by multimodal MRI. J. Cereb. Blood Flow. Metab. Off. J. Int. Soc. Cereb. Blood Flow. Metab. 41, 380–396. 10.1177/0271678X20912364 PMC7812517 32237952

[B47] Kazumata K. Tanaka N. Ishikawa T. Kuroda S. Houkin K. Mitsumori K. (1996). Dissociation of vasoreactivity to acetazolamide and hypercapnia. Comparative study in patients with chronic occlusive major cerebral artery disease. Stroke 27, 2052–2058. 10.1161/01.STR.27.11.2052 8898815

[B48] Kety S. S. Schmidt C. F. (1948). The nitrous oxide method for the quantitative determination of cerebral blood flow in man: theory, procedure and normal values. J. Clin. Invest. 27, 476–483. 10.1172/JCI101994 16695568 PMC439518

[B49] Kim D. Hughes T. M. Lipford M. E. Craft S. Baker L. D. Lockhart S. N. (2021). Relationship between cerebrovascular reactivity and cognition among people with risk of cognitive decline. Front. Physiol. 12, 645342. 10.3389/fphys.2021.645342 34135768 PMC8201407

[B50] Kleinschmidt A. Steinmetz H. Sitzer M. Merboldt K.-D. Frahm J. (1995). Magnetic resonance imaging of regional cerebral blood oxygenation changes under acetazolamide in carotid occlusive disease. Stroke 26, 106–110. 10.1161/01.STR.26.1.106 7839378

[B51] Lattanzi S. Carbonari L. Pagliariccio G. Bartolini M. Cagnetti C. Viticchi G. (2018). Neurocognitive functioning and cerebrovascular reactivity after carotid endarterectomy. Neurology 90, e307–e315. 10.1212/WNL.0000000000004862 29282326

[B52] Le L. N. N. Wheeler G. J. Blockley N. P. Fan A. P. (2023). “Quantitative BOLD with variational bayesian inference: model comparisons with monte carlo simulations and in an elderly cohort,” in ISMRM, Toronto, Canada, 03-08 June 2023.

[B53] Lee H. Englund E. K. Wehrli F. W. (2018). Interleaved quantitative BOLD: combining extravascular r2ʹ - and intravascular r2-measurements for estimation of deoxygenated blood volume and hemoglobin oxygen saturation. NeuroImage 174, 420–431. 10.1016/j.neuroimage.2018.03.043 29580967 PMC5949279

[B54] Leenders K. L. Perani D. Lammertsma A. A. Heather J. D. Buckingham P. Jones T. (1990). Cerebral blood flow, blood volume and oxygen utilization: normal values and effect of age. Brain 113, 27–47. 10.1093/brain/113.1.27 2302536

[B55] Li X. Wang D. Auerbach E. J. Moeller S. Ugurbil K. Metzger G. J. (2015). Theoretical and experimental evaluation of multi-band EPI for high-resolution whole brain pCASL Imaging. NeuroImage 106, 170–181. 10.1016/j.neuroimage.2014.10.029 25462690 PMC4337884

[B56] Lin Z. Lim C. Jiang D. Soldan A. Pettigrew C. Oishi K. (2023). Longitudinal changes in brain oxygen extraction fraction (OEF) in older adults: relationship to markers of vascular and alzheimer’s pathology. Alzheimer’s Dementia 19, 569–577. 10.1002/alz.12727 PMC10838398 35791732

[B57] Liu Y. Li S. Tian X. Leung T. W. Liu L. Liebeskind D. S. (2023). Cerebral haemodynamics in symptomatic intracranial atherosclerotic disease: A narrative review of the assessment methods and clinical implications. Stroke Vasc. Neurol.–2023-002333. svn-2023-002333. 10.1136/svn-2023-002333 PMC10800270 37094991

[B58] Makris N. Goldstein J. M. Kennedy D. Hodge S. M. Caviness V. S. Faraone S. V. (2006). Decreased volume of left and total anterior insular lobule in schizophrenia. Schizophr. Res. 83, 155–171. 10.1016/j.schres.2005.11.020 16448806

[B59] Mandell D. M. Han J. S. Poublanc J. Crawley A. P. Stainsby J. A. Fisher J. A. (2008). Mapping cerebrovascular reactivity using blood oxygen level-dependent MRI in patients with arterial steno-occlusive disease: comparison with arterial spin labeling mri. Stroke 39, 2021–2028. 10.1161/STROKEAHA.107.506709 18451352

[B60] Mikl M. Mareček R. Hluštík P. Pavlicová M. Drastich A. Chlebus P. (2008). Effects of spatial smoothing on fMRI group inferences. Magn. Reson. Imaging 26, 490–503. 10.1016/j.mri.2007.08.006 18060720

[B61] Nemoto E. M. Yonas H. Kuwabara H. Pindzola R. R. Sashin D. Meltzer C. C. (2004). Identification of hemodynamic compromise by cerebrovascular reserve and oxygen extraction fraction in occlusive vascular disease. J. Cereb. Blood Flow. Metab. 24, 1081–1089. 10.1097/01.WCB.0000125887.48838.37 15529008

[B62] Ni W. Christen T. Zun Z. Zaharchuk G. (2015). Comparison of R2′ measurement methods in the normal brain at 3 tesla. Magn. Reson. Med. 73, 1228–1236. 10.1002/mrm.25232 24753286 PMC4308575

[B63] Ni W. W. Christen T. Rosenberg J. Zun Z. Moseley M. E. Zaharchuk G. (2017). Imaging of cerebrovascular reserve and oxygenation in Moyamoya disease. J. Cereb. Blood Flow. Metab. 37, 1213–1222. 10.1177/0271678X16651088 27207169 PMC5453445

[B64] Nicoll D. Lu C. M. Pignone M. McPhee S. J. (2012). Pocket guide to diagnostic tests. 6. McGraw Hill: Access Medicine. Available at: https://accessmedicine.mhmedical.com/content.aspx?bookid=503&sectionid=43474712.

[B65] Olman C. A. Davachi L. Inati S. (2009). Distortion and signal loss in medial temporal lobe. PLOS ONE 4, e8160. 10.1371/journal.pone.0008160 19997633 PMC2780716

[B66] Pinheiro J. C. Bates D. M. (1996). Unconstrained parametrizations for variance-covariance matrices. Stat. Comput. 6, 289–296. 10.1007/BF00140873

[B67] Pinto J. Chappell M. A. Okell T. W. Mezue M. Segerdahl A. R. Tracey I. (2020). Calibration of arterial spin labeling data—Potential pitfalls in post-processing. Magn. Reson. Med. 83, 1222–1234. 10.1002/mrm.28000 31605558 PMC6972489

[B68] Richiardi J. Monsch A. U. Haas T. Barkhof F. Van de Ville D. Radü E. W. (2015). Altered cerebrovascular reactivity velocity in mild cognitive impairment and Alzheimer’s disease. Neurobiol. Aging 36, 33–41. 10.1016/j.neurobiolaging.2014.07.020 25146454

[B69] Robb W. H. Khan O. A. Ahmed H. A. Li J. Moore E. E. Cambronero F. E. (2022). Lower cerebral oxygen utilization is associated with Alzheimer’s disease-related neurodegeneration and poorer cognitive performance among apolipoprotein E ε4 carriers. J. Cereb. Blood Flow. Metab. Off. J. Int. Soc. Cereb. Blood Flow. Metab. 42, 642–655. 10.1177/0271678X211056393 PMC9051148 34743630

[B70] Rolfe D. F. Brown G. C. (1997). Cellular energy utilization and molecular origin of standard metabolic rate in mammals. Physiol. Rev. 77, 731–758. 10.1152/physrev.1997.77.3.731 9234964

[B71] Silvestrini M. Pasqualetti P. Baruffaldi R. Bartolini M. Handouk Y. Matteis M. (2006). Cerebrovascular reactivity and cognitive decline in patients with Alzheimer disease. Stroke 37, 1010–1015. 10.1161/01.STR.0000206439.62025.97 16497984

[B72] Simon A. B. Dubowitz D. J. Blockley N. P. Buxton R. B. (2016). A novel Bayesian approach to accounting for uncertainty in fMRI-derived estimates of cerebral oxygen metabolism fluctuations. NeuroImage 129, 198–213. 10.1016/j.neuroimage.2016.01.001 26790354 PMC4830424

[B73] Smeeing D. P. J. Hendrikse J. Petersen E. T. Donahue M. J. de Vis J. B. (2016). Arterial spin labeling and blood oxygen level-dependent MRI cerebrovascular reactivity in cerebrovascular disease: A systematic review and meta-analysis. Cerebrovasc. Dis. 42, 288–307. 10.1159/000446081 27237626

[B74] Smith S. M. (2002). Fast robust automated brain extraction. Hum. Brain Mapp. 17, 143–155. 10.1002/hbm.10062 12391568 PMC6871816

[B75] Spees W. M. Yablonskiy D. A. Oswood M. C. Ackerman J. J. H. (2001). Water proton MR properties of human blood at 1.5 tesla: magnetic susceptibility, t1, t2, t2*, and non-lorentzian signal behavior. Magn. Reson. Med. 45, 533–542. 10.1002/mrm.1072 11283978

[B76] Stone A. J. Blockley N. P. (2017). A streamlined acquisition for mapping baseline brain oxygenation using quantitative BOLD. NeuroImage 147, 79–88. 10.1016/j.neuroimage.2016.11.057 27915118

[B77] Stone A. J. Harston G. W. J. Carone D. Okell T. W. Kennedy J. Blockley N. P. (2019). Prospects for investigating brain oxygenation in acute stroke: experience with a non-contrast quantitative bold based approach. Hum. Brain Mapp. 40, 2853–2866. 10.1002/hbm.24564 30860660 PMC6563088

[B78] Sukstanskii A. L. Yablonskiy D. A. (2001). Theory of FID NMR signal dephasing induced by mesoscopic magnetic field inhomogeneities in biological systems. J. Magn. Reson. 151, 107–117. 10.1006/jmre.2001.2363 11444944

[B79] Tam C. W. C. Burton E. J. McKeith I. G. Burn D. J. O’Brien J. T. (2005). Temporal lobe atrophy on MRI in Parkinson disease with dementia: A comparison with alzheimer disease and dementia with lewy bodies. Neurology 64, 861–865. 10.1212/01.WNL.0000153070.82309.D4 15753423

[B80] Tchistiakova E. Crane D. E. Mikulis D. J. Anderson N. D. Greenwood C. E. Black S. E. (2015). Vascular risk factor burden correlates with cerebrovascular reactivity but not resting state coactivation in the default mode network. J. Magn. Reson. Imaging JMRI 42, 1369–1376. 10.1002/jmri.24917 25884110

[B81] Uchida Y. Kan H. Inoue H. Oomura M. Shibata H. Kano Y. (2022). Penumbra detection with oxygen extraction fraction using magnetic susceptibility in patients with acute ischemic stroke. Front. Neurology 13, 752450. 10.3389/fneur.2022.752450 PMC8873150 35222239

[B82] Václavů L. Petr J. Petersen E. T. Mutsaerts H. J. M. M. Majoie C. B. L. Wood J. C. (2020). Cerebral oxygen metabolism in adults with sickle cell disease. Am. J. Hematol. 95, 401–412. 10.1002/ajh.25727 31919876 PMC7155077

[B83] Vestergaard M. B. Iversen H. K. Simonsen S. A. Lindberg U. Cramer S. P. Andersen U. B. (2023). Capillary transit time heterogeneity inhibits cerebral oxygen metabolism in patients with reduced cerebrovascular reserve capacity from steno-occlusive disease. J. Cereb. Blood Flow. Metab. 43, 460–475. 10.1177/0271678X221139084 36369740 PMC9941865

[B84] Vorstrup S. Henriksen L. Paulson O. B. (1984). Effect of acetazolamide on cerebral blood flow and cerebral metabolic rate for oxygen. J. Clin. Invest. 74, 1634–1639. 10.1172/JCI111579 6501565 PMC425340

[B85] Wang K. Smith Z. M. Buxton R. B. Swenson E. R. Dubowitz D. J. (2015). Acetazolamide during acute hypoxia improves tissue oxygenation in the human brain. J. Appl. Physiol. Bethesda Md 119, 1494–1500. 10.1152/japplphysiol.00117.2015 PMC4683345 26472861

[B86] Wang Y. Fellah S. Fields M. E. Guilliams K. P. Binkley M. M. Eldeniz C. (2021). Cerebral oxygen metabolic stress, microstructural injury, and infarction in adults with sickle cell disease. Neurology 97, e902–e912. 10.1212/WNL.0000000000012404 34172536 PMC8408504

[B87] Watchmaker J. M. Juttukonda M. R. Davis L. T. Scott A. O. Faraco C. C. Gindville M. C. (2018). Hemodynamic mechanisms underlying elevated oxygen extraction fraction (OEF) in moyamoya and sickle cell anemia patients. J. Cereb. Blood Flow. Metab. 38, 1618–1630. 10.1177/0271678X16682509 28029271 PMC6125968

[B88] Watts M. E. Pocock R. Claudianos C. (2018). Brain energy and oxygen metabolism: emerging role in normal function and disease. Front. Mol. Neurosci. 11, 216. 10.3389/fnmol.2018.00216 29988368 PMC6023993

[B89] Woolrich M. W. Chiarelli P. Gallichan D. Perthen J. Liu T. T. (2006). Bayesian inference of hemodynamic changes in functional arterial spin labeling data. Magn. Reson. Med. 56, 891–906. 10.1002/mrm.21039 16964610

[B90] Xu F. Liu P. Pekar J. J. Lu H. (2015). Does acute caffeine ingestion alter brain metabolism in young adults? NeuroImage 110, 39–47. 10.1016/j.neuroimage.2015.01.046 25644657 PMC4380776

[B91] Yablonskiy D. A. Haacke E. M. (1994). Theory of NMR signal behavior in magnetically inhomogeneous tissues: the static dephasing regime. Magn. Reson. Med. 32, 749–763. 10.1002/mrm.1910320610 7869897

[B92] Yablonskiy D. A. Sukstanskii A. L. He X. (2013). Blood oxygenation level-dependent (BOLD)-based techniques for the quantification of brain hemodynamic and metabolic properties - theoretical models and experimental approaches. NMR Biomed. 26, 963–986. 10.1002/nbm.2839 22927123 PMC3510357

[B93] Yamaguchi T. Kanno I. Uemura K. Shishido F. Inugami A. Ogawa T. (1986). Reduction in regional cerebral metabolic rate of oxygen during human aging. Stroke 17, 1220–1228. 10.1161/01.STR.17.6.1220 3492786

[B94] Yamauchi H. Okazawa H. Kishibe Y. Sugimoto K. Takahashi M. (2004). Oxygen extraction fraction and acetazolamide reactivity in symptomatic carotid artery disease. J. Neurol. Neurosurg. Psychiatry 75, 33–37. 14707303 PMC1757491

[B95] Yin Y. Shu S. Qin L. Shan Y. Gao J.-H. Lu J. (2021). Effects of mild hypoxia on oxygen extraction fraction responses to brain stimulation. J. Cereb. Blood Flow. Metab. 41, 2216–2228. 10.1177/0271678X21992896 33563081 PMC8393298

[B96] Yin Y. Zhang Y. Gao J.-H. (2018). Dynamic measurement of oxygen extraction fraction using a multiecho asymmetric spin echo (MASE) pulse sequence. Magnetic Reson. Med. 80, 1118–1124. 10.1002/mrm.27078 29315817

[B97] Zhang J. Cho J. Zhou D. Nguyen T. D. Spincemaille P. Gupta A. (2018). Quantitative susceptibility mapping-based cerebral metabolic rate of oxygen mapping with minimum local variance. Magnetic Reson. Med. 79, 172–179. 10.1002/mrm.26657 28295523

