# Supplementary material for: Cortical oxygen extraction fraction using quantitative BOLD MRI and cerebral blood flow during vasodilation
Source: Front Physiol. 2023 Oct 6;14:1231793. doi: 10.3389/fphys.2023.1231793 (PMC10588655; doi:10.3389/fphys.2023.1231793)
Supplement: Supplementary file 1 [file DataSheet1.docx]

Supplementary Material

Cortical oxygen extraction fraction using quantitative BOLD MRI and cerebral blood flow during vasodilation

## Supplementary Figures:


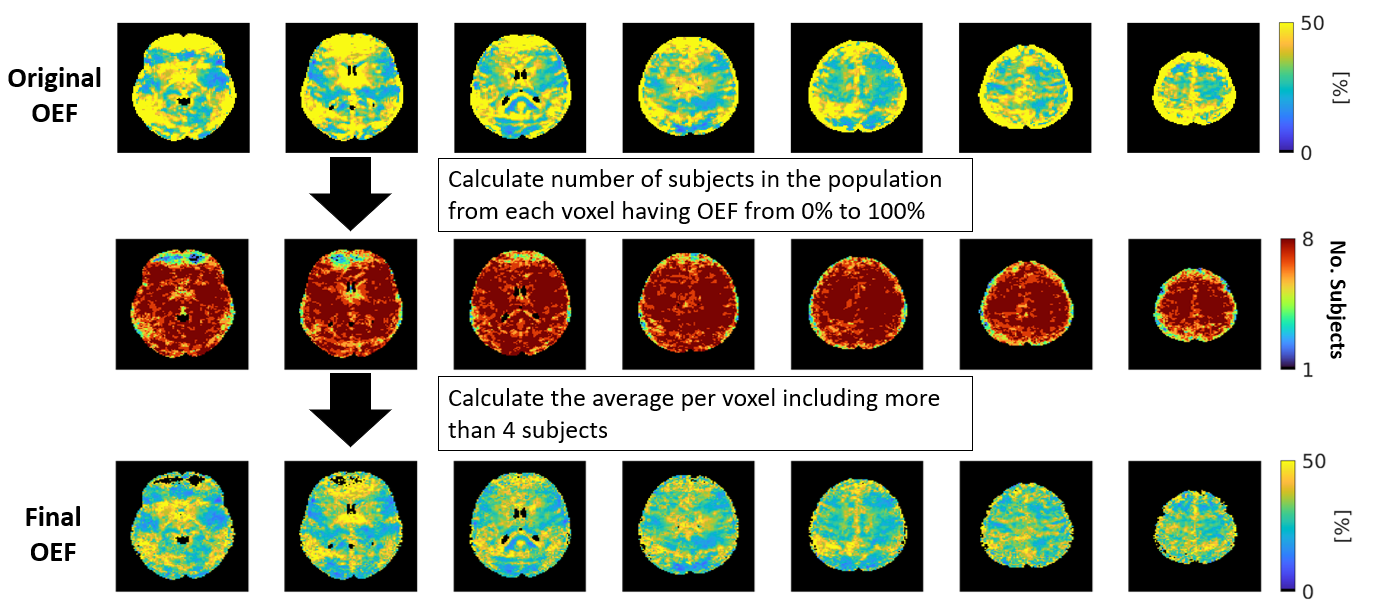


**Supplementary Figure S1.** The complete steps to calculate the average oxygen extraction fraction (OEF) at group level. Original OEF was directly quantified from quantitative BOLD (qBOLD) modeling. First, the total number of subjects with OEF from 0% to 100% from each voxel was calculated. Then, voxels having less than 4 subjects within the physiological range were excluded. Finally, the average OEF was calculated per voxel.


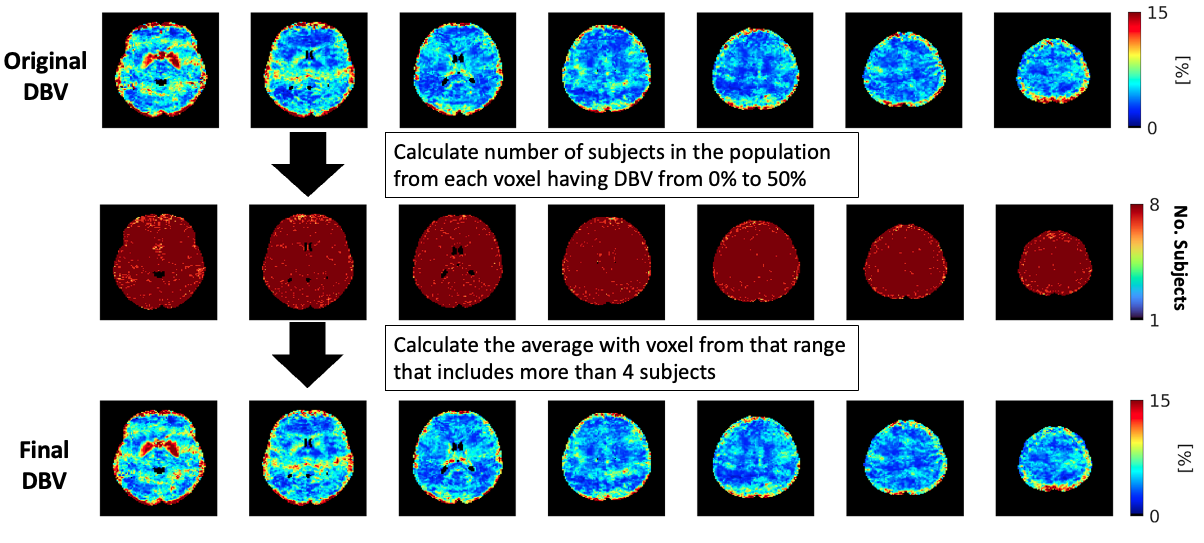


**Supplementary Figure S2.** The complete steps to calculate the average deoxygenated blood volume (DBV) at the group level. Original DBV was directly estimated from asymmetric spin echo (ASE) data with quantitative BOLD (qBOLD) modeling. First, the total number of subjects with DBV from 0% to 50% from each voxel was calculated. Then, voxels having less than 4 subjects within the physiological range were excluded. Finally, the average DBV was calculated per voxel.

**
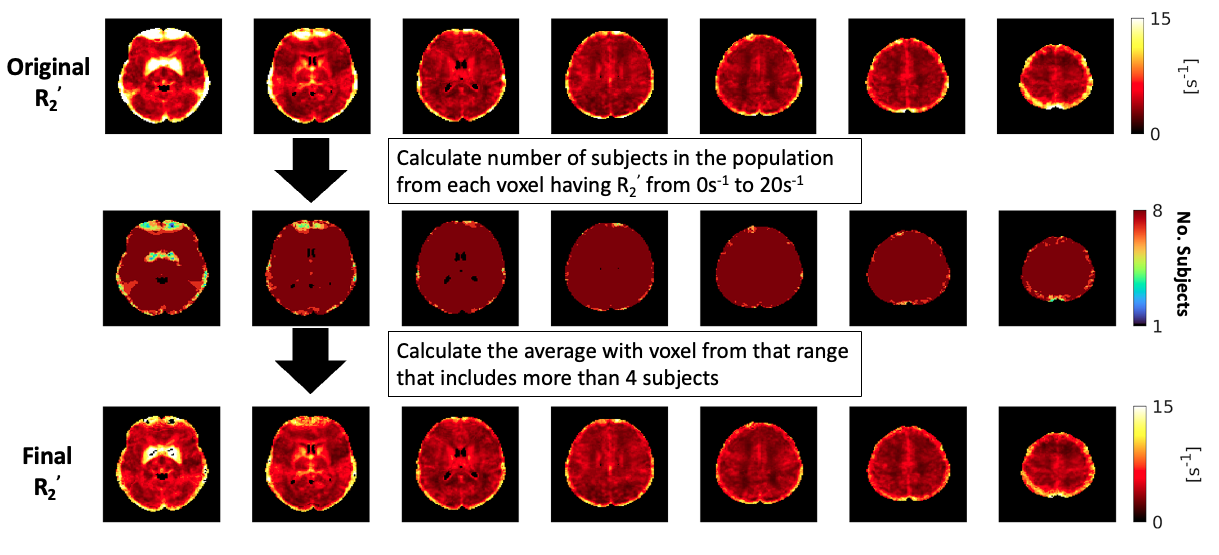
**

**Supplementary Figure S3.** The complete steps to calculate the average irreversible transverse relaxation rate (R_2_^’^) at the group level. Original R_2_^’^was directly estimated from asymmetric spin echo (ASE) data with quantitative BOLD (qBOLD) modeling. First, the total number of subjects with R_2_^’^from 0s^-1^ to 20s^-1^ from each voxel was calculated. Then, voxels having less than 4 subjects within the physiological range were excluded. Finally, the average R_2_^’^was calculated per voxel.


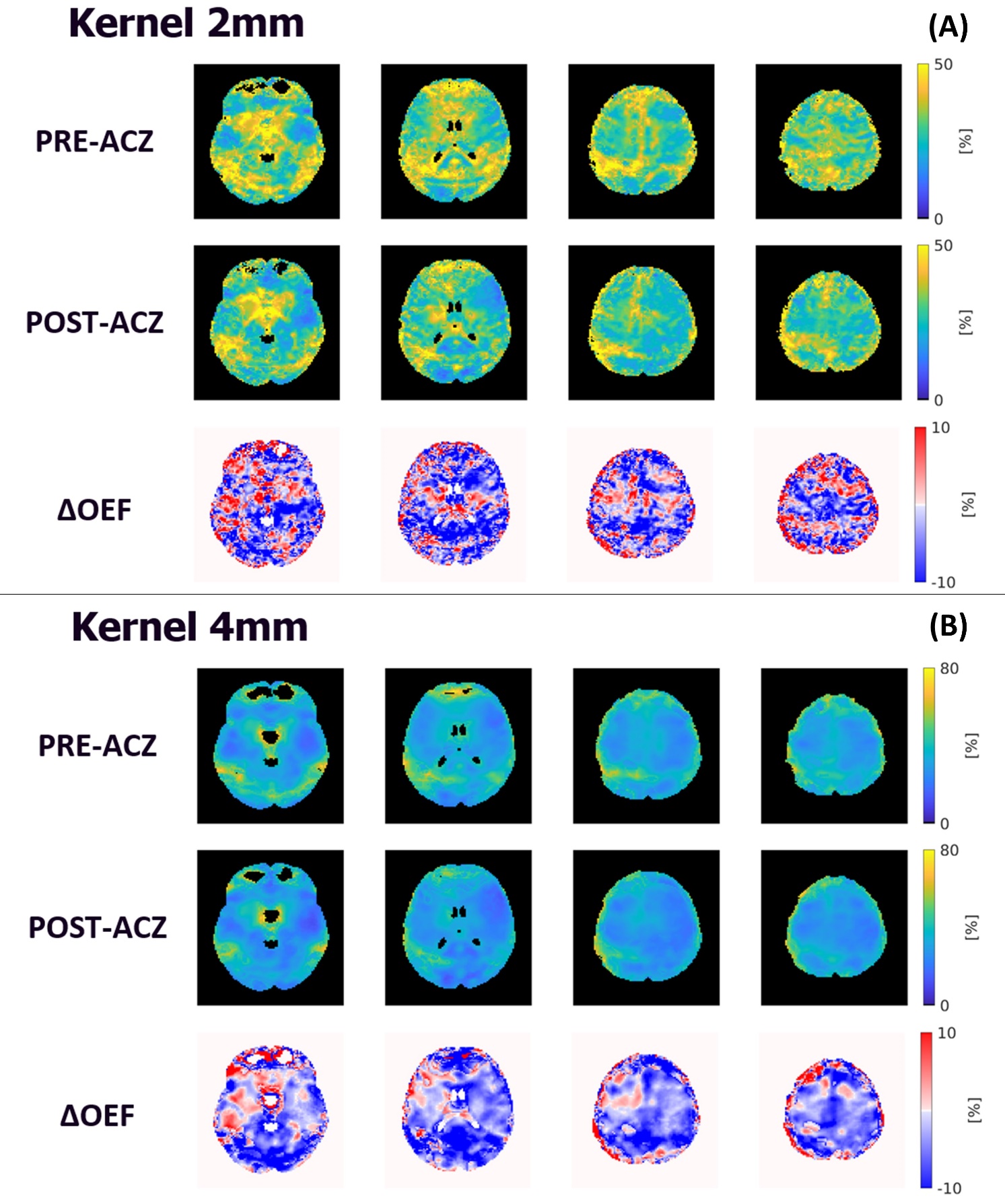


**Supplementary Figure S4.** Oxygen Extraction Fraction (OEF) maps in group average in MNI space before (Pre-ACZ) and after (Post-ACZ) vasodilation (bottom). The difference maps (ΔOEF) between two conditions across all subjects. This figure is generated with Gaussian smoothing kernel of 2mm (A) and 4mm (B).


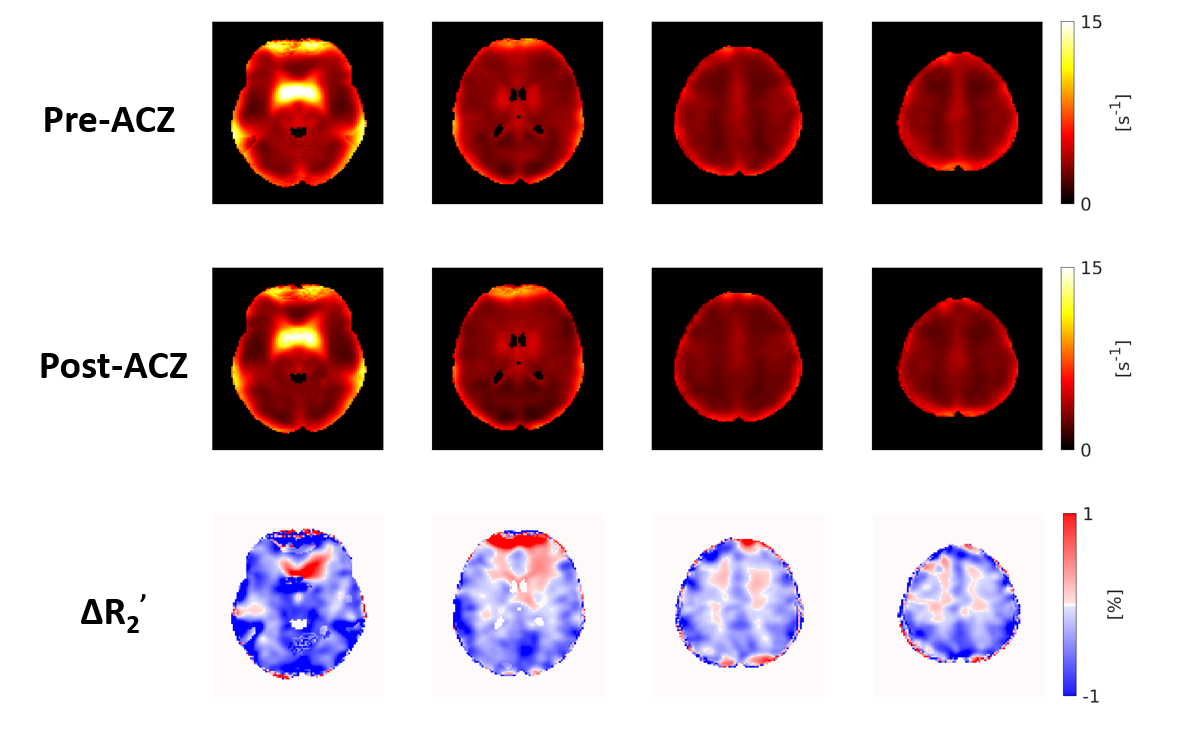


**Supplementary Figure S5.** The group average of transverse relaxation rate (R_2_^’^) (in s^-1^) using monoexponential fitting across all eight healthy subjects before (Pre-ACZ) and after (Post-ACZ) vasodilation with the corresponding absolute difference maps between two conditions (ΔR_2_^’^). All maps were registered to Montreal Neurological Institute (MNI) space.

**Supplementary Table S1.** Percentage of voxels removed (%) due to thresholding in the two conditions before (Pre-ACZ) and after (Post-ACZ) vasodilation for each parameter at chosen kernel of 4mm with chosen prior standard deviation (${\sigma\left( DBV \right)=10}^{\frac{3}{2}}\%; {\sigma\left( R_{2}^{'} \right)=10}^{\frac{1}{2}}s^{-1})$ and with a broader prior standard deviation (${\sigma\left( DBV \right)=10}^{\frac{5}{2}}\%; {\sigma\left( R_{2}^{'} \right)=10}^{\frac{5}{2}}s^{-1})$for variational Bayes framework.

| **Chosen prior standard deviation** | ${\sigma\left( DBV \right)=10}^{\frac{3}{2}}\%; {\sigma\left( R_{2}^{'} \right)=10}^{\frac{1}{2}}s^{-1}$ | |
| --- | --- | --- |
|  | **Pre-ACZ** | **Post-ACZ** |
| **DBV** | 0.21±0.08% | 0.16±0.05% |
| **R_2_^’^** | 2.05±0.67% | 1.84±0.57% |
| **OEF** | 13.1±2.31% | 10.93±1.66% |
|  |  |  |
| **Broader prior standard deviation** | ${\sigma\left( DBV \right)=10}^{\frac{5}{2}}\%; {\sigma\left( R_{2}^{'} \right)=10}^{\frac{5}{2}}s^{-1}$ | |
|  | **Pre-ACZ** | **Post-ACZ** |
| **DBV** | 0.71±0.26% | 0.61±0.24% |
| **R_2_^’^** | 2.14±0.72% | 1.93±0.59% |
| **OEF** | 13.61±2.35% | 11.57±1.85% |

**Supplementary Table S2**. Average transverse relaxation rate (R_2_^’^) (in s^-1^) generated from the monoexponential fitting model before (Pre) and after (Post) vasodilation across all healthy subjects (mean±std) in different regions of interest (ROIs): angular gyrus (AG), medial frontal gyrus (MFG), anterior cingulate gyrus (aCG), posterior cingulate gyrus (pCG), precuneus (PCun), occipital pole (OP), supramarginal gyrus (SG), middle temporal gyrus (MTG), and inferior temporal gyrus (ITG). All tests were performed using a two-sided paired *t*-test with Bonferroni correction. (*p<0.05, significant raw p-value; **p<0.006, significant p-value after correction)

|  | **Pre-ACZ** | **Post-ACZ** | **P-val** |
| --- | --- | --- | --- |
| **AG** | 4.4±0.8 | 3.7±0.7 | 0.118 |
| **MFG** | 6.6±2.4 | 6.2±1.7 | 0.729 |
| **aCG** | 3.5±0.5 | 3.3±0.6 | 0.402 |
| **pCG** | 3.4±0.3 | 2.9±0.4 | 0.028* |
| **PCun** | 3.7±0.7 | 3.0±0.7 | 0.065 |
| **OP** | 4.5±0.4 | 3.9±0.7 | 0.039* |
| **SG** | 5.7±1.2 | 5.0±0.8 | 0.230 |
| **MTG** | 6.1±0.8 | 5.6±0.9 | 0.257 |
| **ITG** | 7.1±1.5 | 7.3±1.7 | 0.748 |
